# Mitochondrial Dysfunction in Cardiac Arrhythmias

**DOI:** 10.3390/cells12050679

**Published:** 2023-02-21

**Authors:** Jielin Deng, Yunqiu Jiang, Zhen Bouman Chen, June-Wha Rhee, Yingfeng Deng, Zhao V. Wang

**Affiliations:** 1 Department of Diabetes and Cancer Metabolism, Beckman Research Institute, City of Hope National Medical Center, Duarte, CA 91010, USA; 2 Irell and Manella Graduate School of Biological Sciences, City of Hope National Medical Center, Duarte, CA 91010, USA; 3 Department of Diabetes Complications and Metabolism, Arthur Riggs Diabetes and Metabolism Research Institute, Beckman Research Institute, City of Hope National Medical Center, Duarte, CA 91010, USA; 4 City of Hope Comprehensive Cancer Center, Duarte, CA 91010, USA; 5 Department of Medicine, Beckman Research Institute, City of Hope National Medical Center, Duarte, CA 91010, USA

**Keywords:** mitochondrial dysfunction, arrhythmia, ATP supply, reactive oxygen species

## Abstract

Electrophysiological and structural disruptions in cardiac arrhythmias are closely related to mitochondrial dysfunction. Mitochondria are an organelle generating ATP, thereby satisfying the energy demand of the incessant electrical activity in the heart. In arrhythmias, the homeostatic supply–demand relationship is impaired, which is often accompanied by progressive mitochondrial dysfunction leading to reduced ATP production and elevated reactive oxidative species generation. Furthermore, ion homeostasis, membrane excitability, and cardiac structure can be disrupted through pathological changes in gap junctions and inflammatory signaling, which results in impaired cardiac electrical homeostasis. Herein, we review the electrical and molecular mechanisms of cardiac arrhythmias, with a particular focus on mitochondrial dysfunction in ionic regulation and gap junction action. We provide an update on inherited and acquired mitochondrial dysfunction to explore the pathophysiology of different types of arrhythmias. In addition, we highlight the role of mitochondria in bradyarrhythmia, including sinus node dysfunction and atrioventricular node dysfunction. Finally, we discuss how confounding factors, such as aging, gut microbiome, cardiac reperfusion injury, and electrical stimulation, modulate mitochondrial function and cause tachyarrhythmia.

## 1. Introduction

Cardiac arrhythmias are defined as disruption in the orderly electrical cycle of excitation and recovery through the myocardium. Arrhythmias can be broadly categorized into tachyarrhythmia and bradyarrhythmia based on the ventricular rate, although there are other classification methods based on the origin, means of propagation, associated symptoms, etc. Arrhythmias are highly heterogenous in pathophysiology and severity and cause substantial morbidity and mortality. Atrial fibrillation (AF) is the most frequent arrhythmia and is associated with an increased risk of stroke and mortality, as well as decreased quality of life [1]. A total of three to six million people in the US suffer from AF, leading to a major healthcare burden [2,3]. Ventricular tachyarrhythmias are the major causes of sudden cardiac death (SCD) in the US, accounting for 80% of cases [4,5]. Bradyarrhythmia and conduction abnormalities can cause syncope and SCD, and patients may also experience fatigue and decreased exercise capacity due to chronotropic incompetence [6].

Treatment of arrhythmias can be divided into medical therapies (e.g., anti-arrhythmic drugs) and electrophysiological interventions (e.g., ablations). The currently available treatment options, however, have limitations. For example, routine medical therapy and catheter AF ablation are often plagued by treatment failures, recurrences, and adverse events. Therefore, basic and translational research is critical to advance our understanding of pathophysiology and ultimately improve arrhythmia management through discovery of novel therapeutics [7].

Cumulative studies showed that mitochondrial dysfunction in cardiomyocytes plays an essential role in arrhythmogenesis in both humans and animal models. Mitochondria are an organelle responsible for the synthesis of adenosine 5′-triphosphate (ATP) via oxidative phosphorylation (OXPHOS) [8]. One-third of the cardiac ATP generated by mitochondria is used for the maintenance of ion channels and transporters, which are imperative for the rhythmic electrical activity of cardiomyocytes. Mitochondrial dysfunction adversely affects aerobic respiration and energy production, leading to impairment in cardiac rhythm. In addition, dysfunctional mitochondria may generate excessive reactive oxygen species (ROS), another factor contributing to ion channel and transporter abnormalities and membrane excitability disturbances, which are all crucial players in the pathogenesis of arrhythmias.

Previous studies demonstrated that mitochondrial dysfunction is associated with both tachyarrhythmia and bradyarrhythmia. Additional evidence points to mitochondrial dysfunction as a causative factor of various arrhythmias. In this review, we summarize pathophysiological relevance of mitochondrial dysfunction in the initiation, development, and progression of arrhythmias, with a focus on underlying molecular mechanisms and potential therapeutic explorations.

## 2. Basic Mechanisms of Arrhythmias

**Figure 1:**
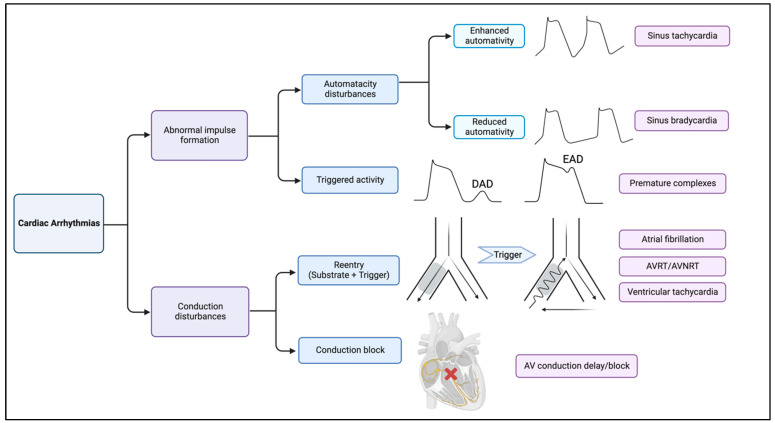
Summary of molecular mechanisms of arrhythmias. Cardiac arrhythmias can be divided to abnormal impulse formation (also termed as focal activity) and conduction disturbances. Automaticity problems involve both enhanced and reduced automaticity, which are the etiology of sinus tachycardia and bradycardia, respectively. Early and late after depolarization is the common mechanism of both atrial and ventricular premature complexes. Reentrant tachyarrhythmias as a conduction disturbance usually involve both substrate (reduced conduction velocity) and trigger where an impulse circles back to re-excite the myocardium through retrograde conduction via the area of functional block. Conduction delay/block also belongs to conduction disturbances.

To better understand how mitochondrial dysfunction promotes cardiac arrhythmias, we first describe the physiologic contributions of ions (e.g., Ca^2+^) to cardiac action potential (AP). Several schemes have been proposed to classify arrhythmias, such as initiation and maintenance factors of arrhythmias [9], cellular or tissue origin of arrhythmias [10], and dynamics-based classification [11]. For example, based on the initiation and maintenance factors, arrhythmias can be categorized into abnormal impulse formation and conduction disturbances: Abnormal impulse formation covers automaticity disturbances and triggered activity, whereas conduction disturbances cover reentry tachycardia and conduction blocks [9] (Figure 1).

### 2.1. Phases of Action Potential (AP)

Cardiac AP results from the sequential opening and closing of ion channel proteins that span the membrane of individual cardiomyocytes [12]. Cardiac AP consists of four phases. Phase zero stands for the rapid depolarization caused by the fast sodium ions (*I_Na_*) diffusing down their electrochemical gradient from the extracellular space, across the membrane, and into the cell. Phase one of AP represents the early rapid repolarization resulting from activation of the fast and slow transient outward potassium currents (*I_K_*). This is followed by a prolonged plateau mediated by a dynamic balance between the inward currents by voltage-gated L-type calcium channel (*I_CaL_*) and Na^+^-Ca^2+^ exchanger (NCX) and the outward currents by the rapid and slow potassium currents (*I_Kr_* and *I_Ks_*, respectively) [13]. This plateau represents phase two of AP. As Ca^2+^ channels become inactivated, the outward potassium currents dominate, causing further repolarization, which is responsible for phase three of AP, and the time-dependent K^+^ current (*I_K1_*) may be the principal current responsible for the final repolarization [14]. In phase four, the Na^+^/K^+^ pump extrudes Na^+^ that has entered during depolarization and restores the K^+^ lost during repolarization.

### 2.2. Abnormal Impulse Formation

#### 2.2.1. Automaticity Disturbances

Automaticity is the spontaneous depolarization caused by a net inward current during phase four of AP. Automaticity is a property resulting from both voltage- and Ca^2+^-dependent mechanisms, which is intrinsic to the sinoatrial node (SAN), the atria, the atrioventricular node (AVN), the His bundle, and the Purkinje fiber network. The voltage-dependent mechanism involves the funny current (*I_f_*), carried by both Na^+^ and K^+^, through hyperpolarization-activated and cyclic nucleotide-gated (HCN) channels located at the plasma membrane. The Ca^2+^-dependent mechanism (Ca^2+^ clock) involves the rhythmic release of Ca^2+^ from the sarcoplasmic reticulum (SR), with subsequent reuptake of Ca^2+^ by SR Ca^2+^-ATPase (SERCA) and extrusion via NCX. Normal automaticity allows cardiomyocytes to generate spontaneous AP, whereas abnormal automaticity includes both enhanced and decreased automaticity. Enhanced automaticity of pacemaker cells can increase the rate of AP discharge through steepening phase four which leads to tachyarrhythmia (e.g., sinus tachycardia, atrial tachycardia, accelerated AV junctional tachycardia), whereas decreased automaticity can lead to bradyarrhythmia (e.g., sinus bradycardia) [9]. If cells do not normally possess the automaticity to obtain this property, premature ectopic heartbeats may occur [9]. Alterations in HCN channel-mediated *I_f_* and Ca^2+^ clock may induce abnormal impulse formation to facilitate associated arrhythmias.

#### 2.2.2. Triggered Activity

Triggered activity, including early afterdepolarizations (EADs) and delayed afterdepolarizations (DADs), is an impulse initiation disturbance that can evoke trains of APs. EADs are caused by net inward currents in phases two/three of AP, induced by *I_CaL_* and NCX [13]. EADs may also be caused by enhanced late sodium current [15]. EADs act as a repolarization interruption and can cause lethal ventricular arrhythmias in the context of action potential duration (APD) prolongation, such as long QT syndrome [16]. DADs usually occur in the context of Ca^2+^ overload, where Ca^2+^ is spontaneously released from SR after repolarization. Ca^2+^ efflux then exits the cell in exchange for Na^+^, generating a net inward depolarizing current [17,18]. The amplitude of DADs increases along with decreasing cycle length, thereby leading to triggered activity. Details in Ca^2+^ homeostasis will be discussed later in this review.

### 2.3. Conduction Disturbances

#### 2.3.1. Reentry Tachycardia

Reentry is a self-sustaining cardiac rhythm abnormality in which AP propagates in a manner analogous to a closed-loop circuit. Reentry is a disorder of impulse conduction where a structural or functional obstacle around which an electrical activity can circulate is required, making reentry distinct from disorders of impulse generation [19]. The substrate of reentry is usually the areas of reduced conduction velocity and APD dispersion. Cardiac conduction velocity is largely determined by the maximum rate of membrane depolarization (dV/dt max) and physical properties of cardiomyocytes, along with their interconnections. Correspondingly, reduced conduction velocity has been attributed to alterations in Na^+^ channel and gap junction function, as well as fibrotic changes, providing substrates for reentry arrhythmias. Reentry is an electrophysiologic mechanism responsible for majority of clinically important arrhythmias [20]. Included among these arrhythmias are AF, atrial flutter, atrioventricular (AV) nodal reentry tachycardia (AVNRT), AV reentry tachycardia (AVRT) involving an accessory pathway, ventricular tachycardia (VT) involving ventricular scars, and ventricular fibrillation (VF). Liu et al. [16] showed that DADs could trigger premature ventricular complexes (PVCs) and cause reentry in vulnerable tissues with areas of unidirectional conduction block, called triggered activity with reentry, a classic example of reentry initiation.

#### 2.3.2. Conduction Block

In addition to reentry, heart block also belongs to conduction abnormalities and can happen anywhere along the cardiac conduction system, including the SAN, the AVN, and the bundle branches. Herein, we mainly discuss ion alterations of conduction disturbance happening in the AVN, also called AV block. AV block is caused by alterations in the ion channel expression of the AVN [13]. As mentioned before, *I_f_* mediated by HCN channels responsible for phase four depolarization, and Ca^2+^ clock is considered related to SAN pacemaking. Recent studies emphasized the importance of HCN channels [21] and *I_f_* within the AVN [22] for AV conduction. In addition, knockout of voltage-dependent Ca^2+^ channels [23] was reported to slow or block AV conduction [21,24]. Although it is not well characterized, the Ca^2+^ clock is considered to control AV conduction [25].

## 3. Mitochondrial Function and Dysfunction

Cardiomyocytes rely on OXPHOS in mitochondria to generate majority of cellular ATP (80–90%). Fatty acids are the main and preferred energetic substrates for ATP production in cardiac muscle. On the other hand, supply of ATP from glycolysis is restricted in the normal heart. When energetic demands increase, however, the relative contribution of glucose utilization increases for glycolytic ATP production. Glucose is converted into pyruvate in the glycolytic pathway, which is a substrate for ATP synthesis in mitochondria. Both fatty acids and glucose can produce acetyl-CoA to enter the TCA cycle, where nicotinamide adenine dinucleotide (NADH) and flavin adenine dinucleotide (FADH2) are produced [26]. NADH and FADH2 serve as electron donors for the mitochondrial electron transport chain (ETC), including Complexes I–IV, as well as the electron transporters ubiquinone and cytochrome c. There are two electron transport pathways in the ETC: Complex I/III/IV with NADH as the substrate; Complex II/III/IV with FADH2 as the substrate [27]. As electrons flow through the ETC, protons travel across the inner membrane from the mitochondrial matrix into the intermembrane space, establishing the proton gradient and the strongly negative mitochondrial membrane potential, ΔΨm. The energy accumulated in the proton gradient is used by Complex V (ATP synthase) to produce ATP [27]. Impaired OXPHOS leads to mitochondrial dysfunction primarily due to defects in ETC enzymes (Complexes I–V) [28]. In addition to producing ATP, mitochondria also generate ROS as a byproduct of OXPHOS: A small part of electrons do not follow the normal transfer order but instead leak out of the ETC and directly interact with O_2_ to generate ROS [29]. Since OXPHOS is not completely coupled, mitochondrial uncoupling is defined as the dissociation between ΔΨm generation and its use for mitochondria-dependent ATP synthesis. Mild uncoupling can be a feedback mechanism to prevent excessive ROS in mitochondria [30]. However, severe mitochondrial uncoupling may cause rapid cellular ATP depletion and excessive ROS production, leading to mitochondrial dysfunction [31]. Mitochondrial ETC proteins are encoded by both mitochondrial DNA (mtDNA) and nuclear DNA (nDNA). mtDNA encodes 13 major respiratory chain proteins with the rest of them encoded by nDNA, 2 ribosomal RNAs, and 22 transfer RNAs. In addition, mitochondria have other functions, including fatty acid oxidation, regulation of Ca^2+^ homeostasis, and cell death, as well as redox control, mostly carried out by nDNA-encoded proteins [32].

Mitochondrial dysfunction may be attributed to acquired factors, including aging, imbalance of gut microbiome, various diseases, adverse effects of drugs and infections, and inheritable factors such as mutations in mtDNA and nDNA (Table 1). All these adverse changes may lead to abnormalities inside mitochondria, with the ultimate outcome of mitochondrial dysfunction with diminished ATP production and excessive ROS generation. Elevated levels of ROS can inhibit the activities of ETC complexes, redox enzymes, and TCA cycle enzymes [32], which further exacerbates ROS production in a vicious circle (ROS-induced ROS-release, RIRR) [33]. Moreover, mitochondria-derived ROS can affect neighboring mitochondria and other organelles, finally propagating the surge of ROS to the whole cell, which is how mitochondrial function deteriorates from a pathophysiological perspective [34].

## 4. Mitochondrial Dysfunction in Arrhythmogenic Pathogenesis

Mitochondria-derived ATP can be used by sarcolemmal and organellar ion channels and transporters, which are required for the electrical activity of cardiac cells. On the other hand, excessive ROS can impact ion currents by modulating the expression of these channels or altering their post-translational modifications. Therefore, mitochondrial dysfunction (decreased ATP and increased ROS) can deteriorate cardiac electrical function, impair intracellular ion homeostasis and membrane excitability, and elicit inflammatory signaling, thus facilitating arrhythmias. Moreover, other mitochondria-associated proteins such as uncoupling proteins (UCPs), mitochondrial connexin (Cx) proteins, mitochondrial renin–angiotensin system (RAS), mitochondria-derived peptides (MDPs), and mitochondrial GPCR kinases (GRKs) and β-arrestins, can regulate mitochondrial function and contribute to the development of arrhythmias (see below).

### 4.1. Sarcolemmal and Intracellular Ion Balance (Ca^2+^, Na^+^, K^+^)

**Figure 2:**
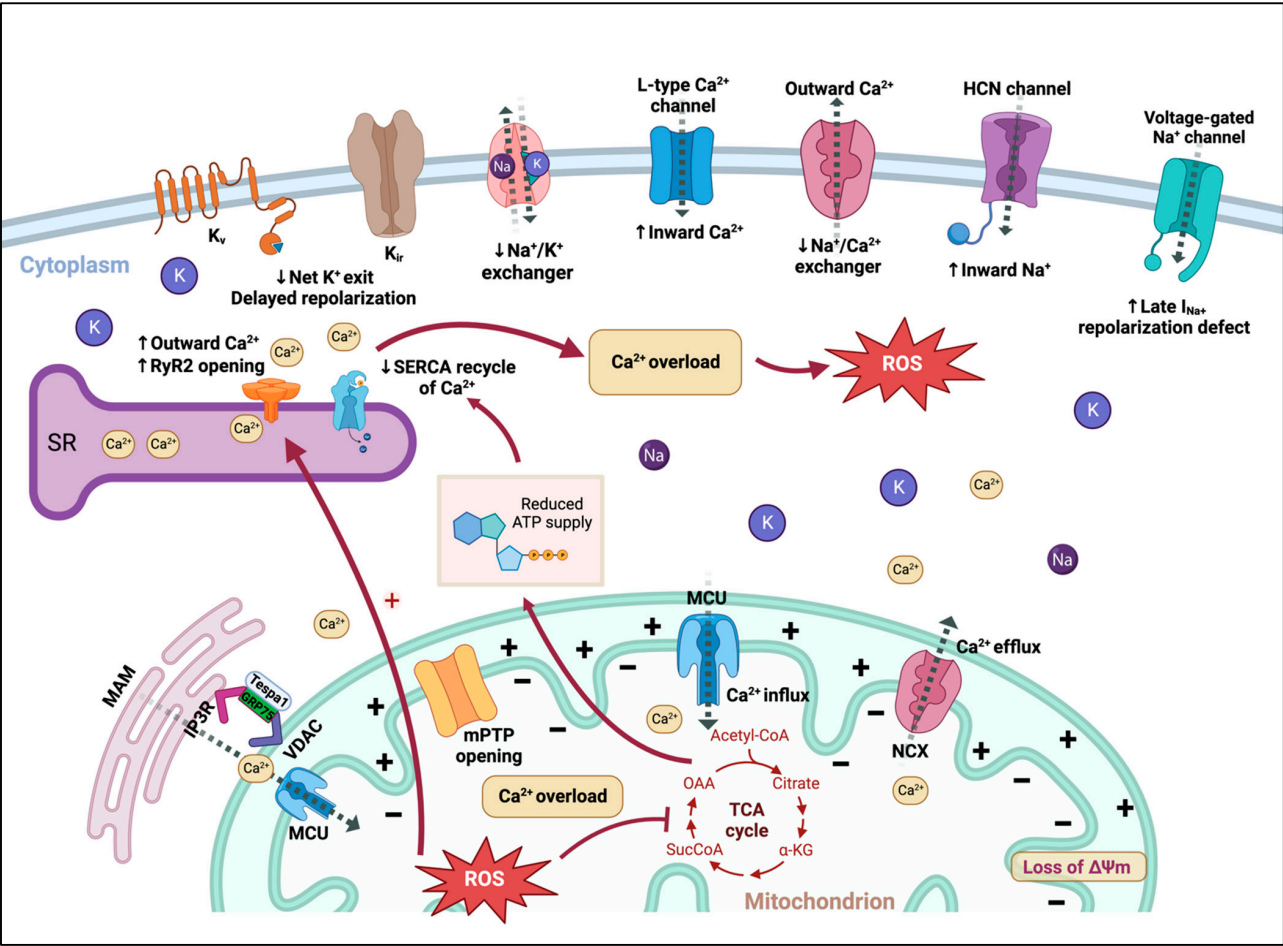
Holistic overview of sarcolemmal and intracellular ion balances in cardiomyocytes and their alterations under mitochondrial dysfunction. Sarcoplasmic reticulum (SR) oversees Ca^2+^ storage and releasing into the cytosol. The main Ca^2+^ release channel in cardiomyocytes is RyR2 while SERCA recycles Ca^2+^ during diastole. ROS increase RyR opening and reduce SERCA recycling via decreased ATP production, thereby causing Ca^2+^ overload. Increased inward Na^+^ from HCN channel can reverse the Na^+^/Ca^2+^ exchanger activity and subsequently lead to intracellular Ca^2+^ overload and arrhythmias. Moreover, under excessive ROS generation, a net decrease in K^+^ exit along with increased inward late Na^+^ current can cause delayed repolarization and prolonged action potential duration (APD). Sarcoplasmic reticulum mitochondrial contacts (SRMCs) are another important location of calcium transport regulation. The IP3R/GRP75/VDAC complex interacts with MCU to relocate Ca^2+^ into the mitochondria matrix. Brown dashed arrows represent directions of ion currents; black solid arrows represent increased or suppressed channel activities.

A holistic overview of sarcolemmal and intracellular ion balance in cardiomyocytes and alterations under mitochondrial dysfunction can be found in Figure 2.

#### 4.1.1. Ca^2+^

Cycling of Ca^2+^ in cardiomyocytes begins with the entry of Ca^2+^ into cells through voltage-gated Ca^2+^ channels, including L-type (*I_Ca-L_*) and T-type (*I_Ca-T_*). *I_Ca-L_* channel is the predominant Ca^2+^ channel in cardiomyocytes participating in myocardial contraction, whereas *I_Ca-T_* channel is mainly expressed in pacemaker cells [46]. The main SR Ca^2+^ release channel in cardiomyocytes is ryanodine receptor 2 (RyR2). Its opening after a small initial amount of Ca^2+^ entry via Ca^2+^ channels results in sarcomere contraction. During the diastolic phase, around 70% of total cytosolic Ca^2+^ is taken up into SR by SERCA [47]. Ca^2+^ extrusion by NCX lowers intracellular Ca^2+^ and counterbalances the entry of Ca^2+^ through sarcolemmal Ca^2+^ channels. Furthermore, communication between SR and mitochondria impacts their functionality in a bidirectional manner [48]. Flux of Ca^2+^ in and out of mitochondria is essential for ATP generation during the constantly varying workloads of the heart by stimulating OXPHOS and increasing NADH production via activation of Ca^2+^-sensitive enzymes in the TCA cycle [49,50]. Mitochondrial Ca^2+^ influx is mainly mediated by the mitochondrial Ca^2+^ uniporter (MCU) complex on the inner mitochondrial membrane [51]. MCU complex is not the only Ca^2+^ transporter in mitochondria. Mitochondrial RyR1 [52], NCX, mitochondrial HCX leucine zipper EF hand-containing transmembrane protein 1 (LETM1) [53], transient receptor potential canonical 3 (TRPC3) [54], uncoupling proteins 2 and 3 (UCP2/3), and other Ca^2+^ transporters [55] are also present in the inner mitochondrial membrane. Mitochondrial Ca^2+^ efflux is primarily mediated by mitochondrial NCX [56,57]. In addition, mitochondrial HCX [53] and mPTP [58,59] are also implicated in mitochondrial Ca^2+^ efflux.

Cardiomyocyte Ca^2+^ homeostasis is strongly influenced by cellular metabolism. Increased cellular ROS are known to cause a net increase in intracellular Ca^2+^ in cardiomyocytes [60]. However, it is still controversial whether the activity of NCX is promoted or inhibited by ROS [61,62,63]. The effects of ROS on *I_Ca-L_* in cardiomyocytes are also under debate [64,65]. Besides sarcolemmal Ca^2+^ channels, excessive mitochondria-derived ROS lead to increased opening of RyR2, which triggers RyR2 Ca^2+^ sparks and increases Ca^2+^ leak from SR [66]. Increased RyR2 activity can, in turn, modulate mitochondrial Ca^2+^ handling, promote mitochondrial ROS emission, and alter channel activity in a pro-arrhythmic feedback cycle [67]. In contrast to RyR2, the activity of SERCA is inhibited by increased oxidative stress [68], which may be attributed to decreased ATP supply for SERCA, secondary to mitochondrial dysfunction [69]. High levels of ROS can also modulate the activity of mitochondrial Ca^2+^-related proteins [70], leading to mitochondrial Ca^2+^ overload, which favors opening of the mPTP and inner membrane anion channel (IMAC) to influence ΔΨm [71,72] and increases RIRR function and ROS production [73]. As mentioned before, ΔΨm depolarization reflects the decreased capacity of mitochondrial ATP production. In addition, excessive mitochondrial ROS can cause oxidative damage to ETC components, leading to impaired ATP production and increased ETC electron leak that further elevates ROS generation. All these effects can be defined as mitochondrial dysfunction, which is pro-arrhythmic and may cause Ca^2+^ alternans by affecting the capacity of mitochondria to handle Ca^2+^ on a beat-to-beat basis [69].

#### 4.1.2. Na^+^

Cardiac voltage-gated Na^+^ (Nav) channels are critical in membrane excitability of cardiomyocytes by generating the rapid upstroke (phase 0) of AP. In addition, Nav channels, together with cardiac gap junctions, control impulse conduction velocity in the myocardium. In response to increased oxidative stress, the expression and function of Nav1.5 channel can be modified, causing an increase in the late component of sodium current (late I_Na_) in cardiomyocytes, leading to APD prolongation and EADs. Increased intracellular Na^+^ caused by elevated late I_Na_ also reverses NCX activity and subsequently leads to intracellular Ca^2+^ overload [33,74]. In addition, increased late I_Na_-induced repolarization defects promote transmural dispersion of repolarization arrhythmic substrate and spatiotemporal heterogeneity, all of which are arrhythmogenic [75,76]. Cardiac conduction velocity is largely determined by peak sodium current (peak I_Na_) and conducted by Nav1.5 channel. Importantly, mitochondrial dysfunction can also lead to reduced peak I_Na_, resulting in reduced conduction velocity and an increased propensity for reentry arrhythmias.

Na^+^/K^+^ pump generates a transmembrane current by pumping three Na^+^ out and two K^+^ into the cell against their concentration gradients with the consumption of one ATP during AP. As a major ion transporter balancing the trans-sarcolemmal Na^+^ and K^+^ gradient and generating the resting membrane potential, Na^+^/K^+^ pump is prone to ATP insufficiency due to its high energy demand [77,78]. In addition, ATP depletion due to mitochondrial dysfunction may also affect Na^+^ clearance and detriment cellular excitability, which may lead to cardiovascular pathologies such as arrhythmias. On one hand, dysfunction of Na^+^/K^+^ pump represents prolonged APD_90_ and raises the AP plateau [79]. On the other hand, buildup of intracellular Na^+^ hinders the concentration gradient that usually drives NCX. Excessive Na^+^ buildup does not favor the extrusion of Ca^2+^ in exchange for Na^+^ entering [80]. This indirect inhibition of NCX further exacerbates Ca^2+^ overload and contributes to arrhythmia-triggering and other detrimental consequences.

#### 4.1.3. K^+^

There are two main types of Kv channels: transient outward Kv (*Ito*) and delayed rectifier Kv (*I_K_*). Currents classified as *Ito* activate and inactivate rapidly upon membrane depolarization. *Ito* underlies the early (phase one) repolarization of AP, which is mostly attributed to *Ito_1_* [81], whereas *I_K_* currents activate depolarization with variable kinetics and underlie the late (phases two and three) repolarization of AP [82]. Similar to Kv channels, multiple functionally distinct types of Kir channels have been identified. Among the Kir channels expressed in mammalian hearts, *I_K1_* contributes to the terminal phase of repolarization and maintenance of resting membrane potentials in ventricular myocytes [83,84], whereas sarcolemmal ATP-sensitive potassium (sarcK_ATP_) channels-mediated currents (IK_ATP_) play an important role in regulating electrophysiological responses under stresses such as cardiac ischemia [85]. Opening of sarcK_ATP_ channels significantly promotes K^+^ efflux, shortens APD [86,87], and slows or blocks AV propagation [88], thereby promoting arrhythmias. In addition to the plasma membrane, sarcK_ATP_ channels are also present in the mitochondrial membrane. Transient opening of mitochondrial K_ATP_ may allow K^+^ to enter mitochondria and slow down the oscillation of ΔΨm. Under mitochondrial dysfunction-induced oxidative stress, multiple repolarizing potassium currents (*I_to_, I_K_*, and *I_K1_*) are suppressed, which can cause delayed repolarization and prolonged APD. In addition, an altered intracellular ATP/ADP ratio, also a consequence of mitochondrial dysfunction, results in the opening of sarcKATP channels [89]. Further, excessive ROS may also cause mitochondrial Ca^2+^ overload and mitochondrial K_ATP_ channel activation, leading to increased K^+^ influx [90]. Taken together, these effects produce an inwardly rectifying repolarizing K^+^ current and Ca^2+^ alternans, which are capable of slowing or blocking cardiac electrical propagation, thereby fomenting arrhythmias [91,92].

### 4.2. Mitochondria-Associated Proteins

**Figure 3:**
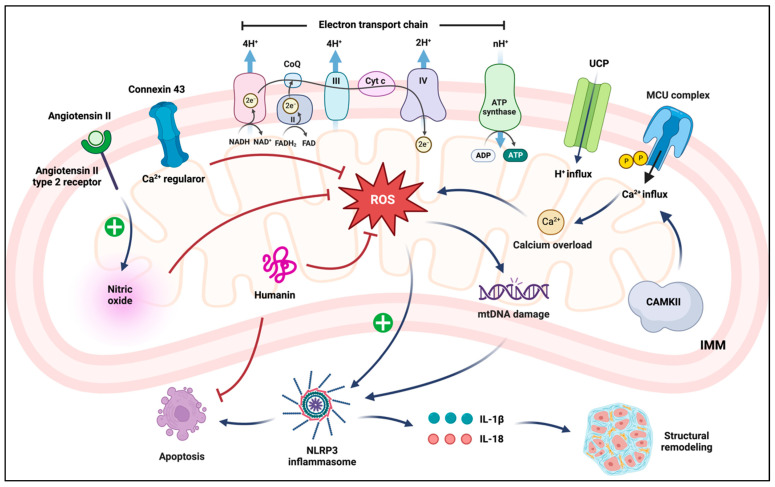
Overview of mitochondrial proteins in cardiac physiology and pathophysiology. A variety of proteins are involved in the functional maintenance of mitochondria. CAMKII activates MCU and allows mitochondrial Ca^2+^ overload, which contributes to ROS production. ROS, along with related mitochondrial DNA damage, contribute to the activation of inflammasomes and subsequent cell death. Structural remodeling induced by inflammatory cytokines produced by inflammasomes follows. On the other hand, the increased level of nitric oxide via activation of angiotensin II type 2 receptor, elevation of mitochondrial connexin located on the inner mitochondrial membrane, and augmentation of humanin peptides all contribute to the mitigation of ROS.

An overview of mitochondrial proteins and their roles in the heart under physiological and pathophysiological conditions can be found in Figure 3.

#### 4.2.1. Mitochondrial Ca^2+^ Transport Proteins

Several Ca^2+^ transport proteins have been described above. Here, we focus on the effects of MCU complex and UCPs in the regulation of mitochondrial Ca^2+^ uptake. Mitochondrial Ca^2+^ uptake is mostly driven by MCU. UCPs are shown to regulate MCU function and, for this reason, are suggested to influence mitochondrial Ca^2+^ handling. Under basal conditions, mitochondrial Ca^2+^ uptake can prevent arrhythmias, but under conditions of Ca^2+^ overload, this action may be pro-arrhythmic.

MCU complex includes pore-forming subunit MCU and auxiliary regulatory proteins MICU1, MICU2, EMRE, MCUb, and MCUR1 [93]. MCU complex has been identified as a highly selective Ca^2+^ channel on the inner mitochondrial membrane [94]. Under pathological conditions, MCU is involved in EAD production [95]. MCU can also cause abnormal repolarization at the cellular level, which is partly dependent on the activation of CaMKII [96]. Knockdown of MCU was reported to inhibit Ca^2+^ uptake in mitochondria, decrease NCX currents, and suppress EADs, thereby reducing arrhythmic risk [95]. At the mechanistic level, ROS accumulation increases MCU activity, leading to mitochondrial Ca^2+^ overload, which enhances the production of mitochondrial ROS, forming a positive feedback loop [97]. On the contrary, another study reported that heart-specific loss of MCU caused defects in both mitochondrial Ca^2+^ uptake and Ca^2+^-induced activation of the TCA cycle, providing both trigger and substrate for arrhythmias [98]. Consistently, Liu et al. found that moderate overexpression of MCU inhibited SR Ca^2+^ leak and thus exerted an anti-arrhythmic effect [99]. The reason for this discrepancy is unclear. We speculate that decreased mitochondrial Ca^2+^ uptake may either promote or inhibit arrhythmias, depending on the severity of heart failure. The precise role of MCU in arrhythmias warrants further study and clarification.

UCPs participate in the regulation of mitochondrial Ca^2+^ homeostasis [100] and mitochondrial ROS generation [101], which may be involved in arrhythmic pathophysiology. To date, five UCPs, including UCP1-5, have been identified in the form of dimers on the inner mitochondrial membrane in mammals [102]. UCP2 and UCP3 uncouple oxidative phosphorylation and reduce ROS production [101,103]. UCP2 and UCP3 also belong to a superfamily of mitochondrial ion transporters [104] and have been reported in Ca^2+^ regulation through MCU-related channel mCa1 and arrhythmia induction [105]. UCP2 overexpression markedly inhibits mitochondrial Ca^2+^ uptake, and UCP2 knockout mice were shown to have a higher susceptibility to arrhythmias with decreased APD after *I_Ca-L_* activation and disturbed Ca^2+^ homeostasis [106]. However, another study suggests that UCP2 upregulation has a negative effect on mitochondrial Ca^2+^ uptake in excitable cells and disrupts excitation-contraction coupling, which potentially causes arrhythmic initiation [107]. These discrepancies may be attributed to different cardiomyocytes used, one being primary neonatal rat ventricular cardiomyocytes and the other being ventricular cardiomyocytes from young adult mice. On the other hand, UCP3 was shown to protect mitochondria from Ca^2+^ overload and propagation of arrhythmic initiation of calcium-induced calcium release (CICR) [105].

#### 4.2.2. Connexin (Cx) Proteins

Impulse conduction through the heart depends on cell-to-cell electrical coupling mediated by gap junctions. Connexin is key to forming these gap junctions [108]. Connexin plays a crucial role in cardiac impulse conduction through the regulation of cardiac conduction velocity [109]. Connexin 43 (Cx43) is known to form gap junctions in ventricular myocytes at the sarcolemmal level, which may play a role in the crosstalk between mitochondrial dysfunction and arrhythmias. For example, mitochondrial ROS are suggested to affect the function of gap junctions by activating c-Src to replace Cx43 [110]. Several studies reported that downregulation of Cx43 results in abnormal conduction, impaired repolarization, prolonged APD, EADs, and DADs, and increased electrical heterogeneity to facilitate reentry arrhythmias [111].

Cx43 is also involved in mitochondrial function maintenance as hemichannels on the inner mitochondrial membrane of subsarcolemmal cardiomyocytes (mitochondrial Cx43) [112]. Mitochondrial Cx43 is an important Ca^2+^ regulator and has been shown to be involved in cardioprotection by ischemic preconditioning, likely involving decreased ROS formation [113]. Consistently, mitochondrial Cx43 deficiency depolarizes ΔΨ_m_ and increases Ca^2+^ within mitochondria, thereby augmenting Ca^2+^ spark frequency, ROS production, and arrhythmia susceptibility [114]. The underlying mechanism may be attributed to the modulation of mitochondrial K_ATP_, of which the opening promotes ROS generation and downstream pathological signaling [115].

#### 4.2.3. Mitochondrial RAS

A growing amount of evidence suggests that intracellular RAS plays an important role in mammalian cell function and is involved in the pathogenesis of arrhythmias. Notably, Abadir et al. revealed the existence of functional mitochondrial RAS with colocalization of angiotensin II (Ang II) and Ang II type 2 receptor (AT2-R) on the inner mitochondrial membrane [116]. The presence of Ang II receptors, including AT1-R and AT2-R, in mitochondria was also identified in Percoll-purified samples [117]. In the presence of Ang II, mitochondrial AT1-R activation may directly affect ROS production. On the other hand, ROS may be generated through stimulation of mitochondrial respiratory chain activity secondary to AT1-R activation [118,119]. Moreover, mitochondrial AT2-R is functionally associated with nitric oxide (NO) production. AT2-R stimulation increased mitochondrial NO generation, which was mitigated by AT2-R antagonist in isolated mitochondria [116]. These studies indicate that the protective AT2-R-mediated NO generation balances AT1-R-mediated ROS generation. Therefore, mitochondrial AT1-R and AT2-R may play opposing roles in maintaining a balance of mitochondrial function and cell survival. An imbalance in mitochondrial RAS can increase AT1-R-mediated ROS, which may impair cardiac gap junction with further cardiac fibrosis (structural remodeling). In addition, ROS can reduce cardiac gap junction expression and impair repolarization, evidenced by prolonged APD, EADs, and DADs (electrical remodeling) [120]. These two types of remodeling may ultimately lead to cardiac arrhythmias.

#### 4.2.4. MDPs

MDPs are a group of peptides encoded by open reading frames of mtDNA [121]. Recently, it has been demonstrated that MDPs play important roles in cardiovascular disease. However, studies exploring the effects of MDPs on arrhythmias so far are scarce. Humanin, one of the MDPs, is encoded in the 16S rRNA region of mtDNA. Humanin exists not only in the circulating body fluids but also in metabolically active organs, such as the heart. Thummasorn et al. found that humanin levels were decreased in the damaged myocardium at the end of cardiac ischemia/reperfusion (I/R). Importantly, administration of a humanin analog could increase humanin levels in the injured myocardium and reduce mitochondrial dysfunction in rats, as indicated by decreases in ROS production, mitochondrial membrane depolarization, mitochondrial swelling, and I/R-induced arrhythmias [122,123]. These results provided novel insights into MDP-mediated cardiac arrhythmia prevention through improving mitochondrial function.

#### 4.2.5. Mitochondrial GRKs and β-Arrestins

GRKs and GPCR adapter proteins such as GRK2 and β-arrestins are crucial regulators of GPCR signaling and mediate the functional crosstalk between mitochondria and other cellular structures [124]. These proteins may move across different cellular compartments, including mitochondria, and interact with various elements, thus affecting signaling transduction in a GPCR-independent manner. Previous studies have uncovered a key role for GRK2 as a regulator of mitochondrial function [124]. For example, ETC components were shown to be regulated by GRK2, particularly the ATP synthase barrel of Complex V, which is critical for ATP production [125]. Further, mitochondrial GRK2-mediated ATP synthesis may affect ROS production and fatty acid metabolism in failing hearts. In addition, GRK2 is involved in mitochondrial fusion and fission via phosphorylating and activating mitofusins [126]. Although several findings indicate that GRK2 is capable of controlling ATP and ROS generation, metabolic stress, and mitochondrial dynamics, the precise role of GRK2 in arrhythmias remains to be unraveled. β-arrestins can also interfere with key mitochondrial processes such as cell death, ROS production, and respiration [127]. Compared with GRK2, the involvement of β-arrestins in the regulation of mitochondrial function in cardiomyocytes requires more work. Although there have been no reports regarding the role of mitochondrial GRKs and GPCR adapters in cardiac arrhythmias, future studies may target these proteins to explore pro-arrhythmic or anti-arrhythmic effects through the regulation of mitochondrial function.

### 4.3. Inflammatory Signaling

In addition to mitochondria-associated proteins, nucleotide-binding domain and leucine-rich repeat pyrin 3 domain (NLRP3) inflammasome is also implicated in mitochondrial dysfunction to facilitate reentry arrhythmias. In cardiomyocytes, resting NLRP3 localizes to SR, whereas NLRP3 inflammasome activation redistributes NLRP3 to SR and mitochondria [128]. Mitochondrial dysfunction plays an important role in the instigation of NLRP3 inflammasome [129]. For example, excessive mitochondria-derived ROS fuel NLRP3 inflammasomal assembly [130]. Mitochondrial ROS also potentiate the release of oxidized mtDNA, which can trigger the assembly of NLRP3 inflammasome [130]. Consequently, NLRP3 inflammasome, via activation of Caspase 1 and generation of interleukin (IL)-1β/IL-18, may induce fibrosis and cause structural remodeling [131]. Moreover, NLRP3 inflammasome upregulation can produce reentry substrate for AF development and higher frequency of spontaneous SR Ca^2+^ releases, which may cause DADs and trigger ectopic activation [132,133,134].

### 4.4. Mitochondria-Associated ER Membranes (MAMs) 

Mitochondria are in close proximity to the ER, and MAMs are the contact sites of the membrane between mitochondria and the ER, which play an important role in organellar communication, such as transport of ions. In cardiomyocytes, MAM contacts are more specifically defined as SR-mitochondria contacts. MAM function depends on acetylated microtubules to support efficient mitochondrial Ca^2+^ uptake during cardiac contraction and relaxation. Mitochondrial Ca^2+^ is then able to boost activities of the TCA cycle and the ETC to promote ATP production [93,135]. Major Ca^2+^ regulatory proteins known to date include IP3R, GRP75, VDACs, Tespa1, Sig1R, SERCA, and RyRs [136]. Among them, IP3R, GRP75, and VDACs form a complex that facilitates the release of Ca^2+^ from SR, Ca^2+^ transport between the two organelles, and uptake of Ca^2+^ by mitochondria [137]. Tespa1 binds GRP75 to help maintain MAM integrity and affect IP3R/GRP75/VDAC complex function [138]. Sig1R forms complexes with another protein, BiP, to stabilize IP3R from the SR side [139]. The imbalance of MAMs is associated with disrupted microtubules, which may lead to abnormal mitochondrial Ca^2+^ uptake and ATP generation, thus facilitating arrhythmia. Several studies found the important effects of MAMs on the development of AF and SAN dysfunction. For example, Li et al. [140] identified a significant loss of MAMs in experimental and clinical AF, and SAN dysfunction has been reported to be associated with the loss of MAM contacts in SAN, which will be discussed in detail in the next section.

## 5. Mitochondrial Dysfunction in Different Arrhythmias

After describing the molecular and ionic alterations linking mitochondrial dysfunction to cardiac arrhythmias, specific causes, including inherited factors, aging, gut microbiome, and various disease that contribute to mitochondrial dysfunction and related arrhythmias, are discussed below.

### 5.1. mtDNA and nDNA Mutation Associated Arrhythmias

Primary mitochondrial respiratory chain diseases (RCD) are systemic disorders caused by sporadic or inherited mutations in mtDNA or nDNA, which can affect genes encoding respiratory chain proteins, characterized by mitochondrial respiratory chain defects and subsequent energy-metabolism imbalance [141]. mtDNA mutations are the most common cause of RCD in adults, identified in ~70% of patients with impaired OXPHOS [142]. The clinical heterogeneity of mtDNA-based mitochondrial diseases is determined, in part, by the type of mutations (protein-coding genes vs. transfer-RNA vs. mtDNA rearrangement) [143]. Although the symptoms may involve nearly all organs, the most prone tissues are the ones that have a high energy demand, such as the heart and skeletal muscle [144]. Electrocardiogram (ECG) abnormalities are seen in up to 70% of RCD patients [145] who are manifested with conduction disturbances, ventricular pre-excitation, and tachyarrhythmias such as AF and ventricular tachycardia.

Conduction disturbances are common in RCD (about 10%) [6,18,28], and their prevalence increases with age [5]. Kearns–Sayre syndrome (KSS) is a specific type of mitochondrial myopathy, with the most common abnormality being a 4.9 kb deletion from nucleotide positions 8469 to 13,447 of mtDNA. Conduction disturbances are the most common symptom occurring in KSS (84% prevalence), with AV block or bradycardia-related polymorphic ventricular tachycardia (PMVT) being part of the criteria for KSS diagnosis [146,147]. PMVT, principally torsade de pointes (TdP) in the setting of QT prolongation and progression to AV block and cardiac arrest [148], has been described as relatively rare [149,150,151]. KSS has also been reported with isolated, asymptomatic right bundle branch block (RBBB) [152].

Conduction disturbances occur less commonly in other forms of RCD with AV or intra-ventricular conduction disturbances. These patients are mainly reported in association with m.8344A > G and m.3243A > G mutations, which are responsible for most cases of myoclonic epilepsy with ragged-red fibers (MERRF), mitochondrial encephalomyopathy, lactic acidosis, and stroke-like episodes (MELAS) [153,154]. Pre-excitation and Wolff–Parkinson–White syndrome (WPW), which can lead to reentry tachyarrhythmias, are present in 15–20% of RCD patients [6,21,28]. They were first observed in Leber’s hereditary optic neuropathy (LHON) patients [155] and may coexist with a type of cardiomyopathy, left ventricular non-compaction (LVNC) [156]. Supraventricular arrhythmias have also been reported in RCD patients, with AF being the most common type [148]. Different types of RCD as the response to specific mtDNA and nDNA mutations are summarized in Table 1.

In line with the findings from RCD patients, acquired mtDNA and nDNA mutations have also been shown to cause mitochondrial dysfunction and associated arrhythmias in rodent models. For example, using the K320E-TwinkleMyo mouse model with an accelerated accumulation of mtDNA deletions in the heart, Baris et al. detected an increased rate of AV block and spontaneous PVC under stress conditions [157]. In addition, cardiac-specific deletion of mitochondrial transcription factor A (mtTFA), a nDNA-encoded key regulator of mtDNA transcription, induced dilated cardiomyopathy with AV block in mice [158]. Moreover, mice with mitochondrial Complex I subunit Ndufs4 deficiency (Ndufs4^−/−^) developed mitochondrial dysfunction and bradyarrhythmia resembling Leigh syndrome (LS). The underlying molecular mechanisms are related to a reduced NAD^+^/NADH ratio, leading to hyperacetylation of Nav1.5 and subsequent reduction of I_Na_ [159].

### 5.2. SAN Dysfunction and AVN Dysfunction

Since SAN cells are noncontractile and autorhythmic with a high density of mitochondria which are the fuel source for SAN automaticity, alterations in mitochondria or mitochondria-SR connectomics may contribute to SAN dysfunction and associated arrhythmias such as sick sinus syndrome. Heart blocks are variable and include prolonged intraventricular conduction time, bundle branch blocks, and complete AV block, which could cause deaths in 20% of patients [160]. Studies have also shown that heart blocks, especially AVN abnormalities, have been linked to mitochondrial dysfunction.

HCN channel expression is positively related to *I_f_* current and the slow component of *I_K_* current (*I_Ks_*), which are involved in SAN automaticity and AVN function. Several studies showed that mitochondrial dysfunction might play an essential role in the regulation of cardiac automaticity and conduction by modulating HCN channels. Yang et al. found that mitochondrial Trx2 cardiac specific deletion mice decreased HCN4 expression and developed sinus bradycardia and AV block [161]. Since Trx2 counteracts oxidative stress by reducing oxidized proteins and indirectly scavenging ROS, Trx2 cardiac specific deletion increased mitochondria-derived ROS, which may lead to SAN and AVN abnormalities through the regulation of HCN channel expression. In addition, cumulative studies demonstrated that mitochondria regulate SAN’s automaticity through Ca^2+^ handling and energy production. Since mitochondria are in close proximity to SR, microdomains between mitochondria and SR in response to the beat-to-beat rise of intracellular Ca^2+^ may play a crucial role in modulating Ca^2+^ cycling in cardiomyocytes [162]. Mitochondria-SR connectomics in SAN ensures adequate ATP production, which is mediated by the Ca^2+^-regulated cAMP/PKA signaling [163]. A recent study reported that impaired mitochondrial connectomics, either through injury to mitochondria or disruption of their MAMs, can cause SAN dysfunction [164]. Moreover, mitochondria-derived ROS bursts rapidly induce cytosolic Ca^2+^ overload by stimulating RyR2 and inhibiting SERCA, which further exacerbates Ca^2+^ dysregulation and leads to AP triggered by abnormal automaticity [162]. Although aforementioned studies have not examined the role of mitochondria in regulating the Ca^2+^ clock in AVN cells, we speculate that mitochondrial dysfunction may also cause AV block through Ca^2+^ clock, similar to the mechanism in SAN cells.

In addition to the studies showing mitochondrial dysfunction-associated ionic alterations in the promotion of SAN and AVN abnormalities, there are reports focusing on other mitochondrial abnormalities-induced bradycardia or heart block. For example, a recent study reported the construction of ACE8/8 transgenic mice with increased cardiac ACE and Ang II levels (mitochondrial RAS activation). These mice showed less severe bradycardia and conduction block through c-Src tyrosine kinase activation, Cx43 reduction, and the impairment of gap junction conduction [165,166,167]. Peroxisome proliferator-activated receptor γ coactivator-1 (PGC-1) is a crucial nuclear transcription co-activator, including PGC-1α, PGC-1β, and PGC-1 related co-activator [168]. They are the major factors in the transcriptional control of mitochondrial components. Whereas PGC-1α^−/−^ or PGC-1β^−/−^ mice presented mild cardiac dysfunction, double deletion of PGC-1α/β caused neonatal death with bradycardia, heart block, and cardiac dysfunction [169].

### 5.3. Reentrant Tachyarrhythmias

#### 5.3.1. AF

AF is the most common arrhythmia in clinics; however, our understanding of the initiation and maintenance of AF remains poor. In most AF patients, the reentry phenomenon is the main pathological presentation. AF severity usually depends on atrial enlargement and fibrosis (substrate), which are caused by systemic or cardiac disease [170]. Substrate abnormality, together with premature atrial beats (trigger), promotes reentry and initiates AF [171]. However, in rarer situations without significant cardiac morphological change, rapid focal activity from pulmonary veins may be more important as the underlying mechanism to induce lone AF either via electrical remodeling or genetic susceptibility. Here, arrhythmogenic foci may depend on SR Ca^2+^ leak due to RyR2 activation that promotes DADs to induce AF [172]. Moreover, recent genome-wide association studies demonstrated that relatively rare mutants in cardiac K^+^ and Na^+^ channels might be involved in AF pathophysiology [173,174,175,176].

The association between mitochondrial dysfunction and AF has been investigated over past decades. Energetic imbalance in AF patients may lead to mitochondrial dysfunction [177]: Frequent depolarization of the atrial myocardium increases ATP demands. In paroxysmal or short-lasting persistent AF, mitochondria can increase ATP synthesis, but over time the production of ATP decreases. As such, the reduced ATP/AMP ratio can activate adenosine monophosphate protein kinase (AMPK), which shifts the metabolic pathway toward glycolysis, affects sarcK_ATP_, and slows inward Ca^2+^ channels to impair ion homeostasis and modify electrophysiological properties of cardiomyocytes [178,179]. In addition to ATP depletion, mitochondrial dysfunction-induced excessive ROS can oxidize RyR2 of SR, leading to aberrant Ca^2+^ sparks, thus facilitating AF development. The sarcolemmal inward Na^+^ channel can also be oxidized [180,181], which may directly alter cardiomyocytes’ excitability and intercellular coupling and establish the functional background to maintain reentry circuits. In addition to electrophysiological remodeling, mitochondrial dysfunction also leads to structural remodeling by promoting cytokine release, activating fibroblasts, and depositing connective tissues to facilitate the development of arrhythmias [182]. Therefore, a variety of factors can cause mitochondrial dysfunction to induce AF. Here, we mainly focus on the role of novel factors, including burst-pacing, aging, and gut microbiome-associated mitochondrial dysfunction, in the pathophysiology of AF.

##### Electrical Stimulation-Induced AF

Burst pacing has been used to induce short episodes of AF in animals [183,184], which is non-physiological but the most used approach in creating AF models in vivo. A recent study has demonstrated that electrical stimulation regulates mitochondrial function through the increase in ROS production [185]. Bukowska et al. applied human atrial samples to rapid burst pacing to induce AF and found an increased number of swollen and completely disrupted mitochondria. It is demonstrated that Ca^2+^ inward current via *I_Ca-L_* contributing to oxidative stress leads to mitochondrial ultrastructural changes [186]. In a rabbit model of pacing-induced AF, the expression of mtDNA-encoded proteins and transcription factors involved in mitochondrial biogenesis was decreased, and the atrial electrophysiological property APD was shortened [187]. Consistently, Shao et al. found that amelioration of mitochondrial dysfunction can reduce burst pacing-induced AF susceptibility through attenuation of ROS generation, systemic inflammation, and atrial fibrosis [188].

##### Aging-Associated Mitochondrial Dysfunction in AF

AF is the most prevalent aging-related arrhythmia affecting millions of people worldwide [189]. Alterations in mitochondrial function in senescent hearts have been documented. A clear link exists between aging and mitochondrial dysfunction in facilitating AF. There have been various mechanisms by which aging causes the increased incidence of AF, including mtDNA damage, clonal expansion of deleterious mutations in mtDNA, transcriptional downregulation of genes in mitochondrial energetics, and deficiencies in mitochondrial ETC enzymes [190,191], providing substrate for reduced energetic efficiency in senescent human hearts [192]. As such, aging-associated dysfunctional mitochondria result in reduced ATP production and high levels of ROS, which can facilitate AF through structural and electrical remodeling. As mentioned before, PGC-1 plays an important role in controlling the transcription of mitochondrial components. Studies showed that the shortening of telomeres by aging [193] might inhibit PGC-1 and cause mitochondrial dysfunction and a series of reactions, such as oxidative stress and intracellular Ca^2+^ overload, eventually inducing AF [194]. PGC-1α has been suggested as a key molecule of mitochondrial function through the regulation of mitochondrial biogenesis and energy metabolism. PGC-1α is also closely related to oxidative stress and inflammation [195,196]. Serum PGC-1α and ΔΨm were found to be reduced in aging-related AF patients [197]. PGC-1β has high sequence similarity to PGC-1α and is also believed to control mitochondrial oxidative energy metabolism and conduction velocity, revealed by reduced voltage-gated inward Na^+^ currents and gap junctions under PGC-1β deficiency. Young PGC-1β^−/−^ hearts developed electrophysiological features resembling aging hearts, which may explain their increased propensity to AF. Moreover, PGC-1β^−/−^ mice reflecting mitochondrial dysfunction showed reduced atrial Cx protein expression and increased cardiac fibrosis, associated with a pro-arrhythmic phenotype progressing with age [198].

##### Gut Microbiota-Associated Mitochondrial Dysfunction in AF

Recent studies have reported that altered intestinal flora composition and fermentation metabolites are implicated in arrhythmias, especially AF. Mitochondria are suggested as the most responsive organelle to microbiotic signaling [199]. A growing amount of evidence shows that gut microbiota can interact with mitochondria in a variety of ways. Moreover, gut microbiome has emerged as a dynamic and central regulator of mitochondrial function in immune and epithelial cells located in the intestine and has been shown to regulate key transcriptional co-activators, transcription factors, and enzymes involved in mitochondrial biogenesis [200]. In addition, gut microbiome signaling to mitochondria has been shown to alter mitochondrial metabolism, which can induce inflammasome signaling [201]. For example, mitochondrial alterations such as increased mitochondrial ROS, oxidized mtDNA, extracellular ATP efflux, and ΔΨm loss are emerging as key activators of NLRP3 inflammasome to promote atrial inflammation and fibrosis [128,201]. Not only the gut microbiome itself but also its derived metabolites, including primary bile acids (BAs), TMAO, indoxyl sulfate, LPS, and choline, are implicated in mitochondrial dysfunction and AF development. For example, primary BAs can activate NADPH oxidase, promoting ROS production and inducing ATP release, which results in NLRP3 inflammasome activation. TMAO also leads to oxidative stress and activates NLRP3 inflammatory and TGFb1/Smad3 signaling pathways. Increased mitochondrial ROS are associated with mPTP opening followed by mitochondrial Ca^2+^ disturbances, which leads to electrical remodeling [202] or the release of pro-apoptotic cytochrome c, Apaf-1, Caspase 9, and Caspase 3, causing cardiac fibrosis [203]. The effects of these gut microbiota and their derived metabolites may increase the likelihood of AF-promoting ectopic firing and AF-maintaining reentry to enhance the susceptibility and maintenance of AF.

##### Other Factors Induced AF

Emerging studies showed that direct ion alteration-induced AF models present mitochondrial dysfunction. For example, Wan et al. found that expression of a gain-of-function mutant of Nav1.5 channel causing increased persistent Na^+^ current led to the development of spontaneous and long-lasting episodes of AF in mice, which also exhibited EADs and mitochondrial dysmorphology. All these pathologies could be attenuated by resolving mitochondrial oxidative stress [204]. In addition, Avula et al. showed that transgenic mice with increased persistent Na^+^ current caused both structural (atrial enlargement and fibrosis) and electrophysiological (EADs) remodeling in atria, leading to AF through modulating mitochondrial ROS [205].

#### 5.3.2. Ventricular Arrhythmias (VAs)

SCD can often be the result of VAs, especially VT/VF, which remains one of the most important public health concerns worldwide [206]. As mentioned earlier, reentry, together with increased triggered activity, is the main mechanism of most tachyarrhythmias, including VAs. Cardiac mitochondrial dysfunction-induced reentry and triggering during VAs may share a similar reentry mechanism with AF. VA-associated mitochondrial dysfunction may reduce ATP and energy production and cause the accumulation of ROS, which further contributes to cardiomyocyte damage. Therefore, reentry circuit is maintained in promoting VAs under stress conditions, such as cardiac I/R injury and direct electrical stimulation.

##### Cardiac Ischemia and I/R Injury-Induced VAs

Ischemia-induced ionic alteration may directly facilitate VAs. In cardiac ischemia or during the ischemic period of I/R, impaired SERCA pump function was observed, indicating that Ca^2+^ waves can be induced by impaired SERCA and thus give rise to Ca^2+^ alternans. These alterations can lead to an increased propensity for cardiac arrhythmias such as ventricular reentry and VFs [207]. On the other hand, ischemia-induced ionic alteration can indirectly promote VAs through the regulation of mitochondrial function. Under ischemia conditions, depletion of oxygen and other substrates greatly limits aerobic respiration, causing the cytosol to become acidic. The increase in Na^+^/H^+^ exchange leads to a high level of intracellular Na^+^, which causes the NCX to work in a reverse mode to increase Ca^2+^ uptake and impair ATP synthesis [208]. These two mechanisms together cause a loss of ion homeostasis, stimulation of ROS, mPTP opening, matrix swelling, OMM rupture, and finally, cell death [209]. Upon reperfusion, intracellular pH is normalized, and OXPHOS resumes in reoxygenated mitochondria, resulting in an increase in ROS production [210]. Both in vitro and in vivo studies showed cellular and ionic alteration-induced mitochondrial dysfunction in the pathophysiology of VAs under cardiac I/R. For example, cardiac ischemia and tachypacing-induced VFs could lead to mitochondrial ΔΨm loss [211]. In addition, cardiac I/R decreased the expression of mitochondrial ETC components [212] and downregulated the ADP/oxygen ratio [213], which is related to impaired ion channel function and post-I/R ventricular arrhythmogenesis. Interestingly, cardiac I/R injury is suggested to induce VAs through the regulation of sarcK_ATP_ rather than mitochondrial K_ATP_ [214]. In addition to I/R, ischemia combined with aging, the latter showing a mosaic of normal cells and mitochondrial deficient cells in the heart, also contributes to a higher susceptibility for VAs through regulation of mitochondrial function. For example, Stöckigt et al. showed that aging-related cardiac mitochondrial dysfunction facilitated the occurrence of spontaneous and inducible VAs after cardiac ischemia, which was associated with the increase in phosphorylated Cx43 and slowing of electrical impulse propagation in the infarct area [215].

##### Electrical Stimulation-Induced VAs

In line with I/R-induced VAs, electrically induced VAs have been shown to be related to mitochondrial ultrastructural alterations [216] and mitochondrial dysfunction, such as mPTP opening and downregulation of mitochondrial ETC components COXBIII and ATPS6 [212]. Furthermore, cardiomyocytes were observed to exhibit mitochondrial abnormalities of cytosolic Na^+^ and mitochondrial Ca^2+^ overload during the recovery of spontaneous circulation after electrical stimulation-induced VAs [217]. An in vitro study also demonstrated that electrical stimulation of cardiomyocytes could disturb CaMKII-dependent Ca^2+^ homeostasis and lead to mitochondrial stress, promoting both structural and electrophysiological remodeling and finally facilitating tachycardia-associated SCD [218].

##### Other Factors Induced VA

Heart failure is accompanied by mitochondrial dysfunction. Similar to ischemia, heart failure can also induce ionic or Ca^2+^ transport protein alteration to facilitate VAs directly. In addition to prolonged APD, reduced Ca^2+^ transient, and elevated Na^+^ concentration, the lowered heart rate threshold for the onset of APD alternans is observed in heart failure [219,220]. For example, Pogwizd et al. found enhanced NCX activity-induced abnormal Ca^2+^ handling, DADs, and initiation of VTs in a heart failure model [221]. Heart failure-induced ionic and electrical remodeling can also indirectly promote VAs through the regulation of mitochondrial Ca^2+^ [95], which could be influenced by excessive ROS. Moreover, combined factors such as heart failure following myocardial infarction are also associated with a high incidence of arrhythmias through electrical remodeling. e.g., increasing the heterogeneity of AP repolarization [222]. Other mitochondrial abnormalities, such as mitochondrial RAS activation, can also promote VAs. Sovari et al. demonstrated that RAS activation in the ACE8/8 mouse model could increase mitochondrial ROS production and reduce conduction velocity via downregulation of Cx43 function and expression, which further leads to an increased risk of VAs [215]. Additional studies showed that both MCU alteration-associated insufficient and excessive mitochondrial Ca^2+^ uptake under the context of heart failure or high-fat diet feeding could lead to excessive ROS generation, which plays a major role in VA pathophysiology. For example, a recent study by Liu et al. showed that MCU overexpression in failing hearts reversed heat failure and prevented ectopic VAs by inhibiting mitochondrial ROS-induced SR Ca^2+^ leak [99]. However, another study by Joseph et al. reported that the presence of MCU promoted VAs during high-fat diet feeding, while cardiac-specific deletion of MCU could be protective in a rodent model [96].

#### 5.3.3. Hereditary Muscular Dystrophy-Associated Arrhythmias 

Mitochondrial dysfunction also plays an important role in arrhythmia development in hereditary muscular dystrophies, in particular, Duchenne muscular dystrophy (DMD). Mitochondrial ROS production, as well as mitochondrial Ca^2+^ uptake, is believed to increase in the DMD mdx mouse model, which may contribute to the pathogenesis of cardiac remodeling and then arrhythmia induction [223,224]. The activity of cardiac I*_CaL_*, Cav1.2, determines Ca^2+^ entry in phase two of AP in cardiomyocytes. In mdx cardiomyocytes, Cav1.2 activation is significantly increased [225], which elevates Ca^2+^ influx during AP. In addition, RyR2-mediated Ca^2+^ leak was reported to contribute to VAs in mdx mice [226]. Excessive Ca^2+^ in the cytoplasm and MAMs also increase mitochondrial Ca^2+^ uptake. Dubinin et al. showed that the augmented mitochondrial Ca^2+^ uptake of mdx mice might be due to an increase in the ratio of MCU and MCUb subunits, whereas the elevation of Ca^2+^ efflux from mitochondria in mdx mice may be due to an increased NCLX level [223]. Moreover, cardiomyocyte mitochondria of mdx mice were more resistant to mPTP opening. All these Ca^2+^ overload effects may disturb cardiac electrophysiology, thereby causing arrhythmias in DMD.

## 6. Conclusions and Perspectives

Mitochondrial dysfunction characterized by reduced ATP synthesis and increased ROS production can lead to cellular and ionic malfunction of the heart, including altered automaticity, triggered activity, reentry phenomenon, and conduction block, thereby causing arrhythmias. Mechanistically, mitochondrial dysfunction is closely related to the pathogenesis of arrhythmias through regulation of the activities of sarcolemmal and mitochondrial ion channels for Na^+^, K^+^, and Ca^2+^, thus leading to cardiac electrical remodeling. In addition, mitochondria-associated proteins and inflammasome signaling, including mitochondrial MCU complex, UCPs, Cx, RAS, MDPs, and NLRP3 inflammasome, are involved in mitochondrial dysfunction by triggering both electrical and structural remodeling. Moreover, mitochondrial dysfunction is implicated in the pathophysiology of specific types of arrhythmias, e.g., RCD-associated arrhythmia, SAN and AVN dysfunction, reentry arrhythmia embracing AF, and VAs. Future studies may focus on exploring mitochondria-related mechanisms in the onset and during progression and new treatments for arrhythmias. Taken together, mitochondrial dysfunction plays an essential role in the etiology of various arrhythmias, which may represent a unifying molecular mechanism and a promising target to ameliorate clinical arrhythmias.

## Figures and Tables

**Table 1:** Summary of inherited mitochondrial dysfunction mutations.

Inheritance Pattern	Clinical Syndrome	Mutations	Affected Genes/Proteins in Mitochondria	Arrhythmias Involved	Cardiac Manifestations	Other Systems Involved
Maternally Transmitted	Myoclonic epilepsy with ragged red fibers (MERRF) [35,36]	m.8344 A to G, m.8356 T to C, m.8363 G to A, and m.8361 G to A	MT-TK	Pre-excitation	Dilated and histiocytoid cardiomyopathy	Myoclonus, spasticity, myopathy
Maternally Transmitted	Leber hereditary optic neuropathy [37]	m.3460 G to A, m.11778 G to A, and m.14484 T to C	MT-ND1, MT-ND4, MT-ND4L, or MT-ND6	Sudden death	Dilated cardiac myopathy	Loss of vision
Maternally Transmitted/Sporadic	Neuropathy, ataxia, and retinitis pigmentosa (NARP) [38,39]	m.8993 T to G	MT-ATP6	Conduction block	Cardiomyopathy	Psychomotor retardation, epilepsy, ataxia, neuropathy, and myopathy
Maternally Transmitted	Leigh syndrome (Mt DNA associated subtype) [40]	More than 75 monogenic causes	MT-TL-1, MT-TK, MT-TI	Conduction block	Hypertrophic cardiomyopathy	Psychomotor regression, respiratory failure, muscular and movement disorder (death at young age)
Maternally Transmitted/Sporadic	Mitochondrial encephalomyopathy, lactic acidosis and stroke-like episodes (MELAS) [41]	m.3243 A to G mutation in MT-TL-1	MT-TL1, MT-TK, and MT-TE genes provide instructions for making tRNAs	Pre-excitation, bundle branch block	Dilated/hypertrophic cardiomyopathy	Severe encephalopathy, lactic acidosis, myoclonus
Maternally Transmitted/Sporadic	Kearns–Sayre syndrome [42,43]	4.9 kb Mt DNA deletion (12 genes)/point mutation	Not determined, involved in mitochondrial protein expression and oxidative phosphorylation	Atrioventricular conduction defects	Cardiomyopathy, syncope, Adams–Stokes syndrome, sudden cardiac death	Anemia, myopathy, lactic acidosis, CNS abnormality, endocrine abnormality, renal disease, sensorineural deafness, and retinal involvement
X-Linked Recessive	Barth syndrome [44]	Mutations or deletions of the highly conserved Xq28 tafazzin (TAZ) gene	Tafazzin protein is essential for remodeling of cardiolipin, a principal phospholipid of the inner mitochondrial membrane	Ventricular arrhythmia, sudden cardiac death, prolonged QTc interval	Dilated/hypertrophic cardiomyopathy, endocardial fibroelastosis (EFE), left ventricular non-compaction (LVNC)	Skeletal myopathy, growth delay, neutropenia and increased urinary excretion of 3-methylglutaconic acid (3-MGCA)
***Autosomal Recessive***	Friedreich’s Ataxia[45]	GAA triplet repeat expansion in the first intron Frataxin (FXN) gene, silencing the gene	Frataxin: expressed in the mitochondria of tissue with high metabolic rates, involved in assembly of iron-sulfur clusters	Conduction block, atrial fibrillation, atrial/ventricular tachycardias, ECG repolarization abnormalities	Hypertrophic cardiomyopathy, heart failure	Gait and limb ataxia, dysarthria, loss of lower limb reflexes, optic neuropathy

Abbreviations: CNS, Central nervous system; ECG, Electrocardiography; MT, Mitochondria.

## Data Availability

Not applicable.

## References

[1] Kornej J., Börschel C.S., Benjamin E.J., Schnabel R.B. (2020). Epidemiology of Atrial Fibrillation in the 21st Century: Novel Methods and New Insights. Circ. Res..

[2] Miyasaka Y., Barnes M.E., Gersh B.J., Cha S.S., Bailey K.R., Abhayaratna W.P., Seward J.B., Tsang T.S. (2006). Secular trends in incidence of atrial fibrillation in Olmsted County, Minnesota, 1980 to 2000, and implications on the projections for future prevalence. Circulation.

[3] Go A.S., Hylek E.M., Phillips K.A., Chang Y., Henault L.E., Selby J.V., Singer D.E. (2001). Prevalence of diagnosed atrial fibrillation in adults: National implications for rhythm management and stroke prevention: The AnTicoagulation and Risk Factors in Atrial Fibrillation (ATRIA) Study. JAMA.

[4] Chugh S.S., Reinier K., Teodorescu C., Evanado A., Kehr E., Al Samara M., Mariani R., Gunson K., Jui J. (2008). Epidemiology of sudden cardiac death: Clinical and research implications. Prog. Cardiovasc. Dis..

[5] Roberts-Thomson K.C., Lau D.H., Sanders P. (2011). The diagnosis and management of ventricular arrhythmias. Nat. Rev. Cardiol..

[6] Epstein A.E., DiMarco J.P., Ellenbogen K.A., Estes N.A., Freedman R.A., Gettes L.S., Gillinov A.M., Gregoratos G., Hammill S.C., Hayes D.L. (2013). 2012 ACCF/AHA/HRS focused update incorporated into the ACCF/AHA/HRS 2008 guidelines for device-based therapy of cardiac rhythm abnormalities: A report of the American College of Cardiology Foundation/American Heart Association Task Force on Practice Guidelines and the Heart Rhythm Society. Circulation.

[7] Heijman J., Algalarrondo V., Voigt N., Melka J., Wehrens X.H., Dobrev D., Nattel S. (2016). The value of basic research insights into atrial fibrillation mechanisms as a guide to therapeutic innovation: A critical analysis. Cardiovasc. Res..

[8] Harris D.A., Das A.M. (1991). Control of mitochondrial ATP synthesis in the heart. Biochem. J..

[9] Antzelevitch C., Burashnikov A. (2011). Overview of Basic Mechanisms of Cardiac Arrhythmia. Card Electrophysiol. Clin..

[10] Anumonwo J.M., Pandit S.V. (2015). Ionic mechanisms of arrhythmogenesis. Trends Cardiovasc. Med..

[11] Weiss J.N., Garfinkel A., Karagueuzian H.S., Nguyen T.P., Olcese R., Chen P.S., Qu Z. (2015). Perspective: A dynamics-based classification of ventricular arrhythmias. J. Mol. Cell. Cardiol..

[12] Tse G. (2016). Mechanisms of cardiac arrhythmias. J. Arrhythm..

[13] Temple I.P., Logantha S.J., Absi M., Zhang Y., Pervolaraki E., Yanni J., Atkinson A., Petkova M., Quigley G.M., Castro S. (2016). Atrioventricular Node Dysfunction and Ion Channel Transcriptome in Pulmonary Hypertension. Circ. Arrhythm. Electrophysiol..

[14] Zang Y., Dai L., Zhan H., Dou J., Xia L., Zhang H. (2013). Theoretical investigation of the mechanism of heart failure using a canine ventricular cell model: Especially the role of up-regulated CaMKII and SR Ca^2+^ leak. J. Mol. Cell. Cardiol..

[15] Dai L., Zang Y., Zheng D., Xia L., Gong Y. (2016). Role of CaMKII and PKA in Early Afterdepolarization of Human Ventricular Myocardium Cell: A Computational Model Study. Comput. Math. Methods Med..

[16] Liu C.F., Cheung J.W., Ip J.E., Thomas G., Yang H., Sharma S., Markowitz S.M., Lerman B.B. (2016). Unifying Algorithm for Mechanistic Diagnosis of Atrial Tachycardia. Circ. Arrhythm. Electrophysiol..

[17] Kass R.S., Tsien R.W., Weingart R. (1978). Ionic basis of transient inward current induced by strophanthidin in cardiac Purkinje fibres. J. Physiol..

[18] Zygmunt A.C., Goodrow R.J., Weigel C.M. (1998). INaCa and ICl(Ca) contribute to isoproterenol-induced delayed after depolarizations in midmyocardial cells. Am. J. Physiol..

[19] Goyal A., Basit H., Bhyan P., Zeltser R. (2022). Reentry Arrhythmia. StatPearls.

[20] Gaztañaga L., Marchlinski F.E., Betensky B.P. (2012). Mechanisms of cardiac arrhythmias. Rev. Esp. Cardiol..

[21] Baruscotti M., Bucchi A., Viscomi C., Mandelli G., Consalez G., Gnecchi-Rusconi T., Montano N., Casali K.R., Micheloni S., Barbuti A. (2011). Deep bradycardia and heart block caused by inducible cardiac-specific knockout of the pacemaker channel gene Hcn4. Proc. Natl. Acad. Sci. USA.

[22] Verrier R.L., Sobrado M.F., Pagotto V.P., Kanas A.F., Machado A.D., Varone B.B., Sobrado L.F., Nearing B.D., Zeng D., Belardinelli L. (2013). Inhibition of I(f) in the atrioventricular node as a mechanism for dronedarone’s reduction in ventricular rate during atrial fibrillation. Heart Rhythm..

[23] Marger L., Mesirca P., Alig J., Torrente A., Dubel S., Engeland B., Kanani S., Fontanaud P., Striessnig J., Shin H.S. (2011). Functional roles of Ca(v)1.3, Ca(v)3.1 and HCN channels in automaticity of mouse atrioventricular cells: Insights into the atrioventricular pacemaker mechanism. Channels.

[24] Matthes J., Yildirim L., Wietzorrek G., Reimer D., Striessnig J., Herzig S. (2004). Disturbed atrio-ventricular conduction and normal contractile function in isolated hearts from Cav1.3-knockout mice. Naunyn Schmiedebergs Arch. Pharmacol..

[25] Kim D., Shinohara T., Joung B., Maruyama M., Choi E.K., On Y.K., Han S., Fishbein M.C., Lin S.F., Chen P.S. (2010). Calcium dynamics and the mechanisms of atrioventricular junctional rhythm. J. Am. Coll. Cardiol..

[26] Nolfi-Donegan D., Braganza A., Shiva S. (2020). Mitochondrial electron transport chain: Oxidative phosphorylation, oxidant production, and methods of measurement. Redox Biol..

[27] Zhao R.Z., Jiang S., Zhang L., Yu Z.B. (2019). Mitochondrial electron transport chain, ROS generation and uncoupling (Review). Int. J. Mol. Med..

[28] Shoffner J.M. (2000). Mitochondrial myopathy diagnosis. Neurol. Clin..

[29] Turrens J.F. (2003). Mitochondrial formation of reactive oxygen species. J. Physiol..

[30] Brand M.D. (2000). Uncoupling to survive? The role of mitochondrial inefficiency in ageing. Exp. Gerontol..

[31] Demine S., Renard P., Arnould T. (2019). Mitochondrial Uncoupling: A Key Controller of Biological Processes in Physiology and Diseases. Cells.

[32] Manolis A.S., Manolis A.A., Manolis T.A., Apostolaki N.E., Apostolopoulos E.J., Melita H., Katsiki N. (2021). Mitochondrial dysfunction in cardiovascular disease: Current status of translational research/clinical and therapeutic implications. Med. Res. Rev..

[33] Armoundas A.A., Hobai I.A., Tomaselli G.F., Winslow R.L., O’Rourke B. (2003). Role of sodium-calcium exchanger in modulating the action potential of ventricular myocytes from normal and failing hearts. Circ. Res..

[34] Di Meo S., Reed T.T., Venditti P., Victor V.M. (2016). Role of ROS and RNS Sources in Physiological and Pathological Conditions. Oxid. Med. Cell. Longev..

[35] Ryosuke T., Noriko O., Tsuyoshi S., Angelos T. (2018). Mitochondrial Cardiomyopathy. Current Perspectives on Cardiomyopathies.

[36] Hameed S., Tadi P. (2022). Myoclonic Epilepsy and Ragged Red Fibers. StatPearls.

[37] Yu-Wai-Man P., Griffiths P.G., Hudson G., Chinnery P.F. (2009). Inherited mitochondrial optic neuropathies. J. Med. Genet..

[38] Birtel J., von Landenberg C., Gliem M., Gliem C., Reimann J., Kunz W.S., Herrmann P., Betz C., Caswell R., Nesbitt V. (2022). Mitochondrial Retinopathy. Ophthalmol. Retin..

[39] Lemoine S., Panaye M., Rabeyrin M., Errazuriz-Cerda E., Mousson de Camaret B., Petiot P., Juillard L., Guebre-Egziabher F. (2018). Renal Involvement in Neuropathy, Ataxia, Retinitis Pigmentosa (NARP) Syndrome: A Case Report. Am. J. Kidney Dis..

[40] Lake N.J., Compton A.G., Rahman S., Thorburn D.R. (2016). Leigh syndrome: One disorder, more than 75 monogenic causes. Ann. Neurol..

[41] El-Hattab A.W., Adesina A.M., Jones J., Scaglia F. (2015). MELAS syndrome: Clinical manifestations, pathogenesis, and treatment options. Mol. Genet. Metab..

[42] Yamashita S., Nishino I., Nonaka I., Goto Y.I. (2008). Genotype and phenotype analyses in 136 patients with single large-scale mitochondrial DNA deletions. J. Hum. Genet..

[43] Tsang S.H., Aycinena A.R.P., Sharma T. (2018). Mitochondrial Disorder: Kearns-Sayre Syndrome. Adv. Exp. Med. Biol..

[44] Clarke S.L., Bowron A., Gonzalez I.L., Groves S.J., Newbury-Ecob R., Clayton N., Martin R.P., Tsai-Goodman B., Garratt V., Ashworth M. (2013). Barth syndrome. Orphanet. J. Rare Dis..

[45] Cook A., Giunti P. (2017). Friedreich’s ataxia: Clinical features, pathogenesis and management. Br. Med. Bull..

[46] Grant A.O. (2009). Cardiac ion channels. Circ. Arrhythm. Electrophysiol..

[47] Bassani J.W., Bassani R.A., Bers D.M. (1994). Relaxation in rabbit and rat cardiac cells: Species-dependent differences in cellular mechanisms. J. Physiol..

[48] Salazar-Ramírez F., Ramos-Mondragón R., García-Rivas G. (2020). Mitochondrial and Sarcoplasmic Reticulum Interconnection in Cardiac Arrhythmia. Front. Cell Dev. Biol..

[49] Liu T., O’Rourke B. (2009). Regulation of mitochondrial Ca^2+^ and its effects on energetics and redox balance in normal and failing heart. J. Bioenerg. Biomembr..

[50] Rizzuto R., De Stefani D., Raffaello A., Mammucari C. (2012). Mitochondria as sensors and regulators of calcium signalling. Nat. Rev. Mol. Cell. Biol..

[51] Kirichok Y., Krapivinsky G., Clapham D.E. (2004). The mitochondrial calcium uniporter is a highly selective ion channel. Nature.

[52] Ryu S.Y., Beutner G., Kinnally K.W., Dirksen R.T., Sheu S.S. (2011). Single channel characterization of the mitochondrial ryanodine receptor in heart mitoplasts. J. Biol. Chem..

[53] Jiang D., Zhao L., Clapham D.E. (2009). Genome-wide RNAi screen identifies Letm1 as a mitochondrial Ca^2+^/H^+^ antiporter. Science.

[54] Feng S., Li H., Tai Y., Huang J., Su Y., Abramowitz J., Zhu M.X., Birnbaumer L., Wang Y. (2013). Canonical transient receptor potential 3 channels regulate mitochondrial calcium uptake. Proc. Natl. Acad. Sci. USA.

[55] Bondarenko A.I., Jean-Quartier C., Malli R., Graier W.F. (2013). Characterization of distinct single-channel properties of Ca^2+^ inward currents in mitochondria. Pflugers Arch.

[56] Palty R., Silverman W.F., Hershfinkel M., Caporale T., Sensi S.L., Parnis J., Nolte C., Fishman D., Shoshan-Barmatz V., Herrmann S. (2010). NCLX is an essential component of mitochondrial Na^+^/Ca^2+^ exchange. Proc. Natl. Acad. Sci. USA.

[57] Maack C., Cortassa S., Aon M.A., Ganesan A.N., Liu T., O’Rourke B. (2006). Elevated cytosolic Na^+^ decreases mitochondrial Ca^2+^ uptake during excitation-contraction coupling and impairs energetic adaptation in cardiac myocytes. Circ. Res..

[58] Bernardi P., von Stockum S. (2012). The permeability transition pore as a Ca^2+^ release channel: New answers to an old question. Cell Calcium.

[59] Zoratti M., Szabò I. (1995). The mitochondrial permeability transition. Biochim. Biophys. Acta..

[60] Goldhaber J.I., Ji S., Lamp S.T., Weiss J.N. (1989). Effects of exogenous free radicals on electromechanical function and metabolism in isolated rabbit and guinea pig ventricle. Implications for ischemia and reperfusion injury. J. Clin. Investig..

[61] Eigel B.N., Gursahani H., Hadley R.W. (2004). ROS are required for rapid reactivation of Na^+^/Ca^2+^ exchanger in hypoxic reoxygenated guinea pig ventricular myocytes. Am. J. Physiol. Heart Circ. Physiol..

[62] Hinata M., Matsuoka I., Iwamoto T., Watanabe Y., Kimura J. (2007). Mechanism of Na^+^/Ca^2+^ exchanger activation by hydrogen peroxide in guinea-pig ventricular myocytes. J. Pharmacol. Sci..

[63] Liu T., O’Rourke B. (2013). Regulation of the Na^+^/Ca^2+^ exchanger by pyridine nucleotide redox potential in ventricular myocytes. J. Biol. Chem..

[64] Viola H.M., Arthur P.G., Hool L.C. (2007). Transient exposure to hydrogen peroxide causes an increase in mitochondria-derived superoxide as a result of sustained alteration in L-type Ca^2+^ channel function in the absence of apoptosis in ventricular myocytes. Circ. Res..

[65] Coetzee W.A., Opie L.H. (1992). Effects of oxygen free radicals on isolated cardiac myocytes from guinea-pig ventricle: Electrophysiological studies. J. Mol. Cell. Cardiol..

[66] Cooper L.L., Li W., Lu Y., Centracchio J., Terentyeva R., Koren G., Terentyev D. (2013). Redox modification of ryanodine receptors by mitochondria-derived reactive oxygen species contributes to aberrant Ca^2+^ handling in ageing rabbit hearts. J. Physiol..

[67] Hamilton S., Terentyeva R., Martin B., Perger F., Li J., Stepanov A., Bonilla I.M., Knollmann B.C., Radwański P.B., Györke S. (2020). Increased RyR2 activity is exacerbated by calcium leak-induced mitochondrial ROS. Basic. Res. Cardiol..

[68] Xu K.Y., Zweier J.L., Becker L.C. (1997). Hydroxyl radical inhibits sarcoplasmic reticulum Ca^2+^-ATPase function by direct attack on the ATP binding site. Circ. Res..

[69] Aggarwal N.T., Makielski J.C. (2013). Redox control of cardiac excitability. Antioxid. Redox Signal..

[70] Görlach A., Bertram K., Hudecova S., Krizanova O. (2015). Calcium and ROS: A mutual interplay. Redox Biol..

[71] Zorova L.D., Popkov V.A., Plotnikov E.Y., Silachev D.N., Pevzner I.B., Jankauskas S.S., Babenko V.A., Zorov S.D., Balakireva A.V., Juhaszova M. (2018). Mitochondrial membrane potential. Anal. Biochem..

[72] Morin D., Assaly R., Paradis S., Berdeaux A. (2009). Inhibition of mitochondrial membrane permeability as a putative pharmacological target for cardioprotection. Curr. Med. Chem..

[73] Zorov D.B., Juhaszova M., Sollott S.J. (2014). Mitochondrial reactive oxygen species (ROS) and ROS-induced ROS release. Physiol. Rev..

[74] Mochizuki S., MacLeod K.T. (1997). Effects of hypoxia and metabolic inhibition on increases in intracellular Ca^2+^ concentration induced by Na^+^/Ca^2+^ exchange in isolated guinea-pig cardiac myocytes. J. Mol. Cell. Cardiol..

[75] Thomas G., Killeen M.J., Grace A.A., Huang C.L. (2008). Pharmacological separation of early afterdepolarizations from arrhythmogenic substrate in DeltaKPQ Scn5a murine hearts modelling human long QT 3 syndrome. Acta. Physiol..

[76] Antzelevitch C., Nesterenko V., Shryock J.C., Rajamani S., Song Y., Belardinelli L. (2014). The role of late I Na in development of cardiac arrhythmias. Handb. Exp. Pharmacol..

[77] Le Masson G., Przedborski S., Abbott L.F. (2014). A computational model of motor neuron degeneration. Neuron.

[78] Zang Y., Marder E. (2021). Interactions among diameter, myelination, and the Na/K pump affect axonal resilience to high-frequency spiking. Proc. Natl. Acad. Sci. USA.

[79] Glitsch H.G. (2001). Electrophysiology of the sodium-potassium-ATPase in cardiac cells. Physiol. Rev..

[80] Bers D.M., Barry W.H., Despa S. (2003). Intracellular Na^+^ regulation in cardiac myocytes. Cardiovasc. Res..

[81] Greenstein J.L., Wu R., Po S., Tomaselli G.F., Winslow R.L. (2000). Role of the calcium-independent transient outward current I(to1) in shaping action potential morphology and duration. Circ. Res..

[82] Nerbonne J.M., Kass R.S. (2005). Molecular physiology of cardiac repolarization. Physiol. Rev..

[83] Lopatin A.N., Nichols C.G. (2001). Inward rectifiers in the heart: An update on I(K1). J. Mol. Cell. Cardiol..

[84] Nichols C.G., Lopatin A.N. (1997). Inward rectifier potassium channels. Annu. Rev. Physiol..

[85] Zhuo M.L., Huang Y., Liu D.P., Liang C.C. (2005). KATP channel: Relation with cell metabolism and role in the cardiovascular system. Int. J. Biochem. Cell Biol..

[86] Chang G.J., Su M.J., Wu T.S., Chen W.P., Kuo C.M. (2008). Electromechanical characterization of cinnamophilin, a natural thromboxane A_2_ receptor antagonist with anti-arrhythmic activity, in guinea-pig heart. Br. J. Pharmacol..

[87] Billman G.E. (2008). The cardiac sarcolemmal ATP-sensitive potassium channel as a novel target for anti-arrhythmic therapy. Pharmacol. Ther..

[88] Akar F.G., O’Rourke B. (2011). Mitochondria are sources of metabolic sink and arrhythmias. Pharmacol. Ther..

[89] van Opbergen C.J.M., den Braven L., Delmar M., van Veen T.A.B. (2019). Mitochondrial Dysfunction as Substrate for Arrhythmogenic Cardiomyopathy: A Search for New Disease Mechanisms. Front. Physiol..

[90] Heinen A., Camara A.K., Aldakkak M., Rhodes S.S., Riess M.L., Stowe D.F. (2007). Mitochondrial Ca^2+^-induced K^+^ influx increases respiration and enhances ROS production while maintaining membrane potential. Am. J. Physiol. Cell Physiol.

[91] Gambardella J., Sorriento D., Ciccarelli M., Del Giudice C., Fiordelisi A., Napolitano L., Trimarco B., Iaccarino G., Santulli G. (2017). Functional Role of Mitochondria in Arrhythmogenesis. Adv. Exp. Med. Biol..

[92] Nakaya H. (2014). Role of ATP-sensitive K^+^ channels in cardiac arrhythmias. J. Cardiovasc. Pharmacol. Ther..

[93] Bertero E., Maack C. (2018). Calcium Signaling and Reactive Oxygen Species in Mitochondria. Circ. Res..

[94] Tarasova N.V., Vishnyakova P.A., Logashina Y.A., Elchaninov A.V. (2019). Mitochondrial Calcium Uniporter Structure and Function in Different Types of Muscle Tissues in Health and Disease. Int. J. Mol. Sci..

[95] Xie A., Song Z., Liu H., Zhou A., Shi G., Wang Q., Gu L., Liu M., Xie L.H., Qu Z. (2018). Mitochondrial Ca^2+^ Influx Contributes to Arrhythmic Risk in Nonischemic Cardiomyopathy. J. Am. Heart Assoc..

[96] Joseph L.C., Reyes M.V., Homan E.A., Gowen B., Avula U.M.R., Goulbourne C.N., Wan E.Y., Elrod J.W., Morrow J.P. (2021). The mitochondrial calcium uniporter promotes arrhythmias caused by high-fat diet. Sci. Rep..

[97] Dong Z., Shanmughapriya S., Tomar D., Siddiqui N., Lynch S., Nemani N., Breves S.L., Zhang X., Tripathi A., Palaniappan P. (2017). Mitochondrial Ca^2+^ Uniporter Is a Mitochondrial Luminal Redox Sensor that Augments MCU Channel Activity. Mol. Cell.

[98] Bertero E., Nickel A., Kohlhaas M., Hohl M., Sequeira V., Brune C., Schwemmlein J., Abeßer M., Schuh K., Kutschka I. (2021). Loss of Mitochondrial Ca^2+^ Uniporter Limits Inotropic Reserve and Provides Trigger and Substrate for Arrhythmias in Barth Syndrome Cardiomyopathy. Circulation.

[99] Liu T., Yang N., Sidor A., O’Rourke B. (2021). MCU Overexpression Rescues Inotropy and Reverses Heart Failure by Reducing SR Ca^2+^ Leak. Circ. Res..

[100] Koshenov Z., Oflaz F.E., Hirtl M., Bachkoenig O.A., Rost R., Osibow K., Gottschalk B., Madreiter-Sokolowski C.T., Waldeck-Weiermair M., Malli R. (2020). The contribution of uncoupling protein 2 to mitochondrial Ca^2+^ homeostasis in health and disease-A short revisit. Mitochondrion.

[101] Hass D.T., Barnstable C.J. (2021). Uncoupling proteins in the mitochondrial defense against oxidative stress. Prog. Retin. Eye Res..

[102] Krauss S., Zhang C.Y., Lowell B.B. (2005). The mitochondrial uncoupling-protein homologues. Nat. Rev. Mol. Cell Biol..

[103] Cadenas S. (2018). Mitochondrial uncoupling, ROS generation and cardioprotection. Biochim. Biophys. Acta Bioenerg..

[104] Arsenijevic D., Onuma H., Pecqueur C., Raimbault S., Manning B.S., Miroux B., Couplan E., Alves-Guerra M.C., Goubern M., Surwit R. (2000). Disruption of the uncoupling protein-2 gene in mice reveals a role in immunity and reactive oxygen species production. Nat. Genet..

[105] Motloch L.J., Gebing T., Reda S., Schwaiger A., Wolny M., Hoppe U.C. (2016). UCP3 Regulates Single-Channel Activity of the Cardiac mCa1. J. Membr. Biol..

[106] Larbig R., Reda S., Paar V., Trost A., Leitner J., Weichselbaumer S., Motloch K.A., Wernly B., Arrer A., Strauss B. (2017). Through modulation of cardiac Ca^2+^ handling, UCP2 affects cardiac electrophysiology and influences the susceptibility for Ca^2+^ -mediated arrhythmias. Exp. Physiol..

[107] Turner J.D., Gaspers L.D., Wang G., Thomas A.P. (2010). Uncoupling protein-2 modulates myocardial excitation-contraction coupling. Circ. Res..

[108] Lo C.W. (2000). Role of gap junctions in cardiac conduction and development: Insights from the connexin knockout mice. Circ. Res..

[109] King J.H., Huang C.L., Fraser J.A. (2013). Determinants of myocardial conduction velocity: Implications for arrhythmogenesis. Front. Physiol..

[110] Sovari A.A., Rutledge C.A., Jeong E.M., Dolmatova E., Arasu D., Liu H., Vahdani N., Gu L., Zandieh S., Xiao L. (2013). Mitochondria oxidative stress, connexin43 remodeling, and sudden arrhythmic death. Circ. Arrhythm. Electrophysiol..

[111] Lerner D.L., Yamada K.A., Schuessler R.B., Saffitz J.E. (2000). Accelerated onset and increased incidence of ventricular arrhythmias induced by ischemia in Cx43-deficient mice. Circulation.

[112] Boengler K., Leybaert L., Ruiz-Meana M., Schulz R. (2022). Connexin 43 in Mitochondria: What Do We Really Know About Its Function?. Front. Physiol..

[113] Papp R., Gönczi M., Kovács M., Seprényi G., Végh A. (2007). Gap junctional uncoupling plays a trigger role in the antiarrhythmic effect of ischaemic preconditioning. Cardiovasc. Res..

[114] Satoh W., Sato H., Kumasaka K., Shindoh C., Miura M. (2021). B-PO02-019 Mitochondrial Connexin43 Is Involved in the Occurrence of Arrhythmias with Modulation of Mitochondrial KAtp Channels. Heart Rhythm.

[115] Oldenburg O., Cohen M.V., Yellon D.M., Downey J.M. (2002). Mitochondrial K(ATP) channels: Role in cardioprotection. Cardiovasc. Res..

[116] Abadir P.M., Foster D.B., Crow M., Cooke C.A., Rucker J.J., Jain A., Smith B.J., Burks T.N., Cohn R.D., Fedarko N.S. (2011). Identification and characterization of a functional mitochondrial angiotensin system. Proc. Natl. Acad. Sci. USA.

[117] Parodi-Rullan R., Barreto-Torres G., Ruiz L., Casasnovas J., Javadov S. (2012). Direct renin inhibition exerts an anti-hypertrophic effect associated with improved mitochondrial function in post-infarction heart failure in diabetic rats. Cell Physiol. Biochem..

[118] Escobales N., Nuñez R.E., Javadov S. (2019). Mitochondrial angiotensin receptors and cardioprotective pathways. Am. J. Physiol. Heart Circ. Physiol..

[119] Li X.C., Zhou X., Zhuo J.L. (2020). Evidence for a Physiological Mitochondrial Angiotensin II System in the Kidney Proximal Tubules: Novel Roles of Mitochondrial Ang II/AT_1a_/O_2_^−^ and Ang II/AT_2_/NO Signaling. Hypertension.

[120] Zhao Z., Fefelova N., Shanmugam M., Bishara P., Babu G.J., Xie L.H. (2011). Angiotensin II induces afterdepolarizations via reactive oxygen species and calmodulin kinase II signaling. J. Mol. Cell. Cardiol..

[121] Wu Y., Sun L., Zhuang Z., Hu X., Dong D. (2021). Mitochondrial-Derived Peptides in Diabetes and Its Complications. Front. Endocrinol..

[122] Thummasorn S., Apaijai N., Kerdphoo S., Shinlapawittayatorn K., Chattipakorn S.C., Chattipakorn N. (2016). Humanin exerts cardioprotection against cardiac ischemia/reperfusion injury through attenuation of mitochondrial dysfunction. Cardiovasc. Ther..

[123] Thummasorn S., Shinlapawittayatorn K., Chattipakorn S.C., Chattipakorn N. (2017). High-dose Humanin analogue applied during ischemia exerts cardioprotection against ischemia/reperfusion injury by reducing mitochondrial dysfunction. Cardiovasc. Ther..

[124] Sorriento D., Gambardella J., Fiordelisi A., Iaccarino G., Illario M. (2019). GRKs and β-Arrestins: “Gatekeepers” of Mitochondrial Function in the Failing Heart. Front. Pharmacol..

[125] Ferrero K., Pfleger J.M., Chuprun K., Barr E., Gao E., Koch W.J. (2021). Abstract MP247: Mitochondrial Complex V Is Regulated By GRK2 in the Heart. Circ. Res..

[126] Mishra P., Chan D.C. (2014). Mitochondrial dynamics and inheritance during cell division, development and disease. Nat. Rev. Mol. Cell Biol..

[127] Zhang J., Xiao H., Shen J., Wang N., Zhang Y. (2017). Different roles of β-arrestin and the PKA pathway in mitochondrial ROS production induced by acute β-adrenergic receptor stimulation in neonatal mouse cardiomyocytes. Biochem. Biophys. Res. Commun..

[128] Zhou R., Yazdi A.S., Menu P., Tschopp J. (2011). A role for mitochondria in NLRP3 inflammasome activation. Nature.

[129] Mishra S.R., Mahapatra K.K., Behera B.P., Patra S., Bhol C.S., Panigrahi D.P., Praharaj P.P., Singh A., Patil S., Dhiman R. (2021). Mitochondrial dysfunction as a driver of NLRP3 inflammasome activation and its modulation through mitophagy for potential therapeutics. Int. J. Biochem. Cell Biol..

[130] Yu J.W., Lee M.S. (2016). Mitochondria and the NLRP3 inflammasome: Physiological and pathological relevance. Arch Pharm. Res..

[131] Kelley N., Jeltema D., Duan Y., He Y. (2019). The NLRP3 Inflammasome: An Overview of Mechanisms of Activation and Regulation. Int. J. Mol. Sci..

[132] Heijman J., Muna A.P., Veleva T., Molina C.E., Sutanto H., Tekook M., Wang Q., Abu-Taha I.H., Gorka M., Künzel S. (2020). Atrial Myocyte NLRP3/CaMKII Nexus Forms a Substrate for Postoperative Atrial Fibrillation. Circ. Res..

[133] Yao C., Veleva T., Scott L., Cao S., Li L., Chen G., Jeyabal P., Pan X., Alsina K.M., Abu-Taha I.D. (2018). Enhanced Cardiomyocyte NLRP3 Inflammasome Signaling Promotes Atrial Fibrillation. Circulation.

[134] Liu H., Zhao Y., Xie A., Kim T.Y., Terentyeva R., Liu M., Shi G., Feng F., Choi B.R., Terentyev D. (2021). Interleukin-1β, Oxidative Stress, and Abnormal Calcium Handling Mediate Diabetic Arrhythmic Risk. JACC Basic. Transl. Sci..

[135] Chen Y., Csordás G., Jowdy C., Schneider T.G., Csordás N., Wang W., Liu Y., Kohlhaas M., Meiser M., Bergem S. (2012). Mitofusin 2-containing mitochondrial-reticular microdomains direct rapid cardiomyocyte bioenergetic responses via interorganelle Ca^2+^ crosstalk. Circ. Res..

[136] Wang Y., Zhang X., Wen Y., Li S., Lu X., Xu R., Li C. (2021). Endoplasmic Reticulum-Mitochondria Contacts: A Potential Therapy Target for Cardiovascular Remodeling-Associated Diseases. Front. Cell Dev. Biol..

[137] Szabadkai G., Bianchi K., Várnai P., De Stefani D., Wieckowski M.R., Cavagna D., Nagy A.I., Balla T., Rizzuto R. (2006). Chaperone-mediated coupling of endoplasmic reticulum and mitochondrial Ca^2+^ channels. J. Cell Biol..

[138] Matsuzaki H., Fujimoto T., Tanaka M., Shirasawa S. (2013). Tespa1 is a novel component of mitochondria-associated endoplasmic reticulum membranes and affects mitochondrial calcium flux. Biochem. Biophys. Res. Commun..

[139] Hayashi T., Su T.P. (2007). Sigma-1 receptor chaperones at the ER-mitochondrion interface regulate Ca^2+^ signaling and cell survival. Cell.

[140] Li J., Qi X., Ramos K.S., Lanters E., Keijer J., de Groot N., Brundel B., Zhang D. (2022). Disruption of Sarcoplasmic Reticulum-Mitochondrial Contacts Underlies Contractile Dysfunction in Experimental and Human Atrial Fibrillation: A Key Role of Mitofusin 2. J. Am. Heart Assoc..

[141] DiMauro S., Schon E.A. (2003). Mitochondrial respiratory-chain diseases. N. Engl. J. Med..

[142] Bates M.G., Bourke J.P., Giordano C., d’Amati G., Turnbull D.M., Taylor R.W. (2012). Cardiac involvement in mitochondrial DNA disease: Clinical spectrum, diagnosis, and management. Eur. Heart J..

[143] Macmillan C., Lach B., Shoubridge E.A. (1993). Variable distribution of mutant mitochondrial DNAs (tRNA(Leu[3243])) in tissues of symptomatic relatives with MELAS: The role of mitotic segregation. Neurology.

[144] Majamaa-Voltti K., Turkka J., Kortelainen M.L., Huikuri H., Majamaa K. (2008). Causes of death in pedigrees with the 3243A>G mutation in mitochondrial DNA. J. Neurol. Neurosurg. Psychiatry.

[145] Limongelli G., Tome-Esteban M., Dejthevaporn C., Rahman S., Hanna M.G., Elliott P.M. (2010). Prevalence and natural history of heart disease in adults with primary mitochondrial respiratory chain disease. Eur. J. Heart Fail..

[146] Kabunga P., Lau A.K., Phan K., Puranik R., Liang C., Davis R.L., Sue C.M., Sy R.W. (2015). Systematic review of cardiac electrical disease in Kearns-Sayre syndrome and mitochondrial cytopathy. Int. J. Cardiol..

[147] Behjati M., Sabri M.R., Etemadi Far M., Nejati M. (2021). Cardiac complications in inherited mitochondrial diseases. Heart Fail. Rev..

[148] Finsterer J., Kothari S. (2014). Cardiac manifestations of primary mitochondrial disorders. Int. J. Cardiol..

[149] Oginosawa Y., Abe H., Nagatomo T., Mizuki T., Nakashima Y. (2003). Sustained polymorphic ventricular tachycardia unassociated with QT prolongation or bradycardia in the Kearns-Sayre syndrome. Pacing Clin. Electrophysiol..

[150] Wilmin S., De Bels D., Knecht S., Gottignies P., Gazagnes M.D., Devriendt J. (2012). Torsade de pointes in Kearns-Sayre syndrome. Pract. Neurol..

[151] Nakano T., Imanaka K., Uchida H., Isaka N., Takezawa H. (1987). Myocardial ultrastructure in Kearns-Sayre syndrome. Angiology.

[152] Katsanos K.H., Pappas C.J., Patsouras D., Michalis L.K., Kitsios G., Elisaf M., Tsianos E.V. (2002). Alarming atrioventricular block and mitral valve prolapse in the Kearns-Sayre syndrome. Int. J. Cardiol..

[153] Wahbi K., Larue S., Jardel C., Meune C., Stojkovic T., Ziegler F., Lombès A., Eymard B., Duboc D., Laforêt P. (2010). Cardiac involvement is frequent in patients with the m.8344A>G mutation of mitochondrial DNA. Neurology.

[154] Majamaa-Voltti K., Peuhkurinen K., Kortelainen M.L., Hassinen I.E., Majamaa K. (2002). Cardiac abnormalities in patients with mitochondrial DNA mutation 3243A>G. BMC Cardiovasc. Disord.

[155] Nikoskelainen E.K., Savontaus M.L., Huoponen K., Antila K., Hartiala J. (1994). Pre-excitation syndrome in Leber’s hereditary optic neuropathy. Lancet.

[156] Ichida F. (2009). Left ventricular noncompaction. Circ J.

[157] Baris O.R., Ederer S., Neuhaus J.F., von Kleist-Retzow J.C., Wunderlich C.M., Pal M., Wunderlich F.T., Peeva V., Zsurka G., Kunz W.S. (2015). Mosaic Deficiency in Mitochondrial Oxidative Metabolism Promotes Cardiac Arrhythmia during Aging. Cell Metab..

[158] Wang J., Wilhelmsson H., Graff C., Li H., Oldfors A., Rustin P., Brüning J.C., Kahn C.R., Clayton D.A., Barsh G.S. (1999). Dilated cardiomyopathy and atrioventricular conduction blocks induced by heart-specific inactivation of mitochondrial DNA gene expression. Nat. Genet..

[159] Yoon J.Y., Daneshgar N., Chu Y., Chen B., Hefti M., Vikram A., Irani K., Song L.S., Brenner C., Abel E.D. (2022). Metabolic rescue ameliorates mitochondrial encephalo-cardiomyopathy in murine and human iPSC models of Leigh syndrome. Clin. Transl. Med..

[160] Anan R., Nakagawa M., Miyata M., Higuchi I., Nakao S., Suehara M., Osame M., Tanaka H. (1995). Cardiac involvement in mitochondrial diseases. A study on 17 patients with documented mitochondrial DNA defects. Circulation.

[161] Yang B., Huang Y., Zhang H., Huang Y., Zhou H.J., Young L., Xiao H., Min W. (2020). Mitochondrial thioredoxin-2 maintains HCN4 expression and prevents oxidative stress-mediated sick sinus syndrome. J. Mol. Cell. Cardiol..

[162] Li Q., Su D., O’Rourke B., Pogwizd S.M., Zhou L. (2015). Mitochondria-derived ROS bursts disturb Ca^2+^ cycling and induce abnormal automaticity in guinea pig cardiomyocytes: A theoretical study. Am. J. Physiol. Heart Circ. Physiol..

[163] Yaniv Y., Juhaszova M., Lyashkov A.E., Spurgeon H.A., Sollott S.J., Lakatta E.G. (2011). Ca^2+^-regulated-cAMP/PKA signaling in cardiac pacemaker cells links ATP supply to demand. J. Mol. Cell. Cardiol..

[164] Ren L., Gopireddy R.R., Perkins G., Zhang H., Timofeyev V., Lyu Y., Diloretto D.A., Trinh P., Sirish P., Overton J.L. (2022). Disruption of mitochondria-sarcoplasmic reticulum microdomain connectomics contributes to sinus node dysfunction in heart failure. Proc. Natl. Acad. Sci. USA.

[165] Donoghue M., Wakimoto H., Maguire C.T., Acton S., Hales P., Stagliano N., Fairchild-Huntress V., Xu J., Lorenz J.N., Kadambi V. (2003). Heart block, ventricular tachycardia, and sudden death in ACE2 transgenic mice with downregulated connexins. J. Mol. Cell. Cardiol..

[166] Xiao H.D., Fuchs S., Campbell D.J., Lewis W., Dudley S.C., Kasi V.S., Hoit B.D., Keshelava G., Zhao H., Capecchi M.R. (2004). Mice with cardiac-restricted angiotensin-converting enzyme (ACE) have atrial enlargement, cardiac arrhythmia, and sudden death. Am. J. Pathol..

[167] Yang K.C., Bonini M.G., Dudley S.C. (2014). Mitochondria and arrhythmias. Free Radic. Biol. Med..

[168] Uguccioni G., Hood D.A. (2011). The importance of PGC-1alpha in contractile activity-induced mitochondrial adaptations. Am. J. Physiol. Endocrinol. Metab..

[169] Holgersen E.M., Gandhi S., Zhou Y., Kim J., Vaz B., Bogojeski J., Bugno M., Shalev Z., Cheung-Ong K., Goncalves J. (2021). Transcriptome-Wide Off-Target Effects of Steric-Blocking Oligonucleotides. Nucleic. Acid. Ther..

[170] Vaziri S.M., Larson M.G., Benjamin E.J., Levy D. (1994). Echocardiographic predictors of nonrheumatic atrial fibrillation. The Framingham Heart Study. Circulation.

[171] Khan R. (2004). Identifying and understanding the role of pulmonary vein activity in atrial fibrillation. Cardiovasc. Res..

[172] Voigt N., Heijman J., Wang Q., Chiang D.Y., Li N., Karck M., Wehrens X.H.T., Nattel S., Dobrev D. (2014). Cellular and molecular mechanisms of atrial arrhythmogenesis in patients with paroxysmal atrial fibrillation. Circulation.

[173] Wakili R., Voigt N., Kääb S., Dobrev D., Nattel S. (2011). Recent advances in the molecular pathophysiology of atrial fibrillation. J. Clin. Investig..

[174] Gudbjartsson D.F., Holm H., Gretarsdottir S., Thorleifsson G., Walters G.B., Thorgeirsson G., Gulcher J., Mathiesen E.B., Njølstad I., Nyrnes A. (2009). A sequence variant in ZFHX3 on 16q22 associates with atrial fibrillation and ischemic stroke. Nat. Genet..

[175] Ellinor P.T., Lunetta K.L., Albert C.M., Glazer N.L., Ritchie M.D., Smith A.V., Arking D.E., Müller-Nurasyid M., Krijthe B.P., Lubitz S.A. (2012). Meta-analysis identifies six new susceptibility loci for atrial fibrillation. Nat. Genet..

[176] Dridi H., Kushnir A., Zalk R., Yuan Q., Melville Z., Marks A.R. (2020). Intracellular calcium leak in heart failure and atrial fibrillation: A unifying mechanism and therapeutic target. Nat. Rev. Cardiol..

[177] Wiersma M., van Marion D.M.S., Wüst R.C.I., Houtkooper R.H., Zhang D., Groot N.M.S., Henning R.H., Brundel B. (2019). Mitochondrial Dysfunction Underlies Cardiomyocyte Remodeling in Experimental and Clinical Atrial Fibrillation. Cells.

[178] Yoshida H., Bao L., Kefaloyianni E., Taskin E., Okorie U., Hong M., Dhar-Chowdhury P., Kaneko M., Coetzee W.A. (2012). AMP-activated protein kinase connects cellular energy metabolism to KATP channel function. J. Mol. Cell. Cardiol..

[179] Sasaki N., Sato T., Marbán E., O’Rourke B. (2001). ATP consumption by uncoupled mitochondria activates sarcolemmal K(ATP) channels in cardiac myocytes. Am. J. Physiol. Heart Circ. Physiol.

[180] Liu M., Liu H., Dudley S.C. (2010). Reactive oxygen species originating from mitochondria regulate the cardiac sodium channel. Circ. Res..

[181] Yoo S., Aistrup G., Shiferaw Y., Ng J., Mohler P.J., Hund T.J., Waugh T., Browne S., Gussak G., Gilani M. (2018). Oxidative stress creates a unique, CaMKII-mediated substrate for atrial fibrillation in heart failure. JCI Insight.

[182] Karam B.S., Chavez-Moreno A., Koh W., Akar J.G., Akar F.G. (2017). Oxidative stress and inflammation as central mediators of atrial fibrillation in obesity and diabetes. Cardiovasc. Diabetol..

[183] Shan J., Xie W., Betzenhauser M., Reiken S., Chen B.X., Wronska A., Marks A.R. (2012). Calcium leak through ryanodine receptors leads to atrial fibrillation in 3 mouse models of catecholaminergic polymorphic ventricular tachycardia. Circ. Res..

[184] Schrickel J.W., Bielik H., Yang A., Schimpf R., Shlevkov N., Burkhardt D., Meyer R., Grohé C., Fink K., Tiemann K. (2002). Induction of atrial fibrillation in mice by rapid transesophageal atrial pacing. Basic. Res. Cardiol..

[185] Lyu J.J., Mehta J.L., Li Y., Ye L., Sun S.N., Sun H.S., Li J.C., Zhang D.M., Wei J. (2018). Mitochondrial Autophagy and NLRP3 Inflammasome in Pulmonary Tissues from Severe Combined Immunodeficient Mice after Cardiac Arrest and Cardiopulmonary Resuscitation. Chin. Med. J..

[186] Bukowska A., Schild L., Keilhoff G., Hirte D., Neumann M., Gardemann A., Neumann K.H., Röhl F.W., Huth C., Goette A. (2008). Mitochondrial dysfunction and redox signaling in atrial tachyarrhythmia. Exp. Biol. Med..

[187] Dong J., Zhao J., Zhang M., Liu G., Wang X., Liu Y., Yang N., Liu Y., Zhao G., Sun J. (2016). β3-Adrenoceptor Impairs Mitochondrial Biogenesis and Energy Metabolism during Rapid Atrial Pacing-Induced Atrial Fibrillation. J. Cardiovasc. Pharmacol. Ther..

[188] Shao Q., Meng L., Lee S., Tse G., Gong M., Zhang Z., Zhao J., Zhao Y., Li G., Liu T. (2019). Empagliflozin, a sodium glucose co-transporter-2 inhibitor, alleviates atrial remodeling and improves mitochondrial function in high-fat diet/streptozotocin-induced diabetic rats. Cardiovasc. Diabetol..

[189] Zoni-Berisso M., Lercari F., Carazza T., Domenicucci S. (2014). Epidemiology of atrial fibrillation: European perspective. Clin. Epidemiol..

[190] Muller-Hocker J. (1989). Cytochrome-c-oxidase deficient cardiomyocytes in the human heart—An age-related phenomenon. A histochemical ultracytochemical study. Am. J. Pathol..

[191] Krishnan K.J., Greaves L.C., Reeve A.K., Turnbull D. (2007). The ageing mitochondrial genome. Nucleic. Acids Res..

[192] Emelyanova L., Preston C., Gupta A., Viqar M., Negmadjanov U., Edwards S., Kraft K., Devana K., Holmuhamedov E., O’Hair D. (2018). Effect of Aging on Mitochondrial Energetics in the Human Atria. J. Gerontol. A Biol. Sci. Med. Sci..

[193] Moslehi J., DePinho R.A., Sahin E. (2012). Telomeres and mitochondria in the aging heart. Circ. Res..

[194] Sahin E., Colla S., Liesa M., Moslehi J., Müller F.L., Guo M., Cooper M., Kotton D., Fabian A.J., Walkey C. (2011). Telomere dysfunction induces metabolic and mitochondrial compromise. Nature.

[195] Chandrasekaran K., Anjaneyulu M., Inoue T., Choi J., Sagi A.R., Chen C., Ide T., Russell J.W. (2015). Mitochondrial transcription factor A regulation of mitochondrial degeneration in experimental diabetic neuropathy. Am. J. Physiol. Endocrinol. Metab..

[196] Jeganathan J., Saraf R., Mahmood F., Pal A., Bhasin M.K., Huang T., Mittel A., Knio Z., Simons R., Khabbaz K. (2017). Mitochondrial Dysfunction in Atrial Tissue of Patients Developing Postoperative Atrial Fibrillation. Ann. Thorac. Surg..

[197] Liu C., Bai J., Dan Q., Yang X., Lin K., Fu Z., Lu X., Xie X., Liu J., Fan L. (2021). Mitochondrial Dysfunction Contributes to Aging-Related Atrial Fibrillation. Oxid. Med. Cell Longev..

[198] Edling C.E., Fazmin I.T., Saadeh K., Chadda K.R., Ahmad S., Valli H., Huang C.L., Jeevaratnam K. (2019). Molecular basis of arrhythmic substrate in ageing murine peroxisome proliferator-activated receptor gamma co-activator deficient hearts modelling mitochondrial dysfunction. Biosci. Rep..

[199] Jackson D.N., Theiss A.L. (2020). Gut bacteria signaling to mitochondria in intestinal inflammation and cancer. Gut Microbes.

[200] Clark A., Mach N. (2017). The Crosstalk between the Gut Microbiota and Mitochondria during Exercise. Front. Physiol..

[201] Prochnicki T., Latz E. (2017). Inflammasomes on the Crossroads of Innate Immune Recognition and Metabolic Control. Cell Metab..

[202] Ferko M., Andelová N., Szeiffová Bačová B., Jašová M. (2019). Myocardial Adaptation in Pseudohypoxia: Signaling and Regulation of mPTP via Mitochondrial Connexin 43 and Cardiolipin. Cells.

[203] Gawałko M., Agbaedeng T.A., Saljic A., Müller D.N., Wilck N., Schnabel R., Penders J., Rienstra M., van Gelder I., Jespersen T. (2022). Gut microbiota, dysbiosis and atrial fibrillation. Arrhythmogenic mechanisms and potential clinical implications. Cardiovasc. Res..

[204] Wan E., Abrams J., Weinberg R.L., Katchman A.N., Bayne J., Zakharov S.I., Yang L., Morrow J.P., Garan H., Marx S.O. (2016). Aberrant sodium influx causes cardiomyopathy and atrial fibrillation in mice. J. Clin. Investig..

[205] Avula U.M.R., Dridi H., Chen B.X., Yuan Q., Katchman A.N., Reiken S.R., Desai A.D., Parsons S., Baksh H., Ma E. (2021). Attenuating persistent sodium current-induced atrial myopathy and fibrillation by preventing mitochondrial oxidative stress. JCI Insight.

[206] Krokhaleva Y., Vaseghi M. (2019). Update on prevention and treatment of sudden cardiac arrest. Trends Cardiovasc. Med..

[207] Liu J., Zhao X., Gong Y., Zhang J., Zang Y., Xia L. (2019). Exploring Impaired SERCA Pump-Caused Alternation Occurrence in Ischemia. Comput. Math. Methods Med..

[208] Gazmuri R.J., Radhakrishnan J., Ayoub I.M. (2019). Sodium-Hydrogen Exchanger Isoform-1 Inhibition: A Promising Pharmacological Intervention for Resuscitation from Cardiac Arrest. Molecules.

[209] Jones S.P., Teshima Y., Akao M., Marbán E. (2003). Simvastatin attenuates oxidant-induced mitochondrial dysfunction in cardiac myocytes. Circ. Res..

[210] Acosta D., Ramos K., Li-Goldman C.P. (1984). Cell injury of cultured rat myocardial cells after reoxygenation of hypoxic cultures in the presence and absence of calcium. In Vitro.

[211] Shibayama J., Taylor T.G., Venable P.W., Rhodes N.L., Gil R.B., Warren M., Wende A.R., Abel E.D., Cox J., Spitzer K.W. (2013). Metabolic determinants of electrical failure in ex-vivo canine model of cardiac arrest: Evidence for the protective role of inorganic pyrophosphate. PLoS ONE.

[212] Szendrei L., Turoczi T., Kovacs P., Vecsernyes M., Das D.K., Tosaki A. (2002). Mitochondrial gene expression and ventricular fibrillation in ischemic/reperfused nondiabetic and diabetic myocardium. Biochem. Pharmacol..

[213] García-Rivas Gde J., Carvajal K., Correa F., Zazueta C. (2006). Ru360, a specific mitochondrial calcium uptake inhibitor, improves cardiac post-ischaemic functional recovery in rats in vivo. Br. J. Pharmacol..

[214] Fischbach P.S., White A., Barrett T.D., Lucchesi B.R. (2004). Risk of ventricular proarrhythmia with selective opening of the myocardial sarcolemmal versus mitochondrial ATP-gated potassium channel. J. Pharmacol. Exp. Ther..

[215] Stöckigt F., Beiert T., Knappe V., Baris O.R., Wiesner R.J., Clemen C.S., Nickenig G., Andrié R.P., Schrickel J.W. (2017). Aging-related mitochondrial dysfunction facilitates the occurrence of serious arrhythmia after myocardial infarction. Biochem. Biophys. Res. Commun..

[216] Fang X., Huang Z., Zhu J., Jiang L., Li H., Fu Y., Sun S., Tang W. (2012). Ultrastructural evidence of mitochondrial abnormalities in postresuscitation myocardial dysfunction. Resuscitation.

[217] Wang S., Radhakrishnan J., Ayoub I.M., Kolarova J.D., Taglieri D.M., Gazmuri R.J. (2007). Limiting sarcolemmal Na^+^ entry during resuscitation from ventricular fibrillation prevents excess mitochondrial Ca^2+^ accumulation and attenuates myocardial injury. J. Appl. Physiol..

[218] Geng L., Wang Z., Cui C., Zhu Y., Shi J., Wang J., Chen M. (2018). Rapid Electrical Stimulation Increased Cardiac Apoptosis Through Disturbance of Calcium Homeostasis and Mitochondrial Dysfunction in Human Induced Pluripotent Stem Cell-Derived Cardiomyocytes. Cell Physiol. Biochem..

[219] O’Rourke B., Kass D.A., Tomaselli G.F., Kääb S., Tunin R., Marbán E. (1999). Mechanisms of altered excitation-contraction coupling in canine tachycardia-induced heart failure, I: Experimental studies. Circ. Res..

[220] Zang Y.L., Xia L. (2014). Cellular mechanism of cardiac alternans: An unresolved chicken or egg problem. J. Zhejiang Univ. Sci. B.

[221] Pogwizd S.M., Qi M., Yuan W., Samarel A.M., Bers D.M. (1999). Upregulation of Na^+^/Ca^2+^ exchanger expression and function in an arrhythmogenic rabbit model of heart failure. Circ. Res..

[222] Hegyi B., Bossuyt J., Griffiths L.G., Shimkunas R., Coulibaly Z., Jian Z., Grimsrud K.N., Sondergaard C.S., Ginsburg K.S., Chiamvimonvat N. (2018). Complex electrophysiological remodeling in postinfarction ischemic heart failure. Proc. Natl. Acad. Sci. USA.

[223] Dubinin M.V., Talanov E.Y., Tenkov K.S., Starinets V.S., Mikheeva I.B., Belosludtsev K.N. (2020). Transport of Ca^2+^ and Ca^2+^-dependent permeability transition in heart mitochondria in the early stages of Duchenne muscular dystrophy. Biochim. Biophys. Acta Bioenerg..

[224] Hughes M.C., Ramos S.V., Turnbull P.C., Edgett B.A., Huber J.S., Polidovitch N., Schlattner U., Backx P.H., Simpson J.A., Perry C.G.R. (2020). Impairments in left ventricular mitochondrial bioenergetics precede overt cardiac dysfunction and remodelling in Duchenne muscular dystrophy. J. Physiol..

[225] Koenig X., Rubi L., Obermair G.J., Cervenka R., Dang X.B., Lukacs P., Kummer S., Bittner R.E., Kubista H., Todt H. (2014). Enhanced currents through L-type calcium channels in cardiomyocytes disturb the electrophysiology of the dystrophic heart. Am. J. Physiol. Heart Circ. Physiol..

[226] Fauconnier J., Thireau J., Reiken S., Cassan C., Richard S., Matecki S., Marks A.R., Lacampagne A. (2010). Leaky RyR2 trigger ventricular arrhythmias in Duchenne muscular dystrophy. Proc. Natl. Acad. Sci. USA.

